# Supplementary material for: Transplantation of hPSC-derived pericyte-like cells promotes functional recovery in ischemic stroke mice
Source: Nat Commun. 2020 Oct 15;11:5196. doi: 10.1038/s41467-020-19042-y (PMC7566513; doi:10.1038/s41467-020-19042-y)
Supplement: Supplementary file 1 — Supplementary Information [file 41467_2020_19042_MOESM1_ESM.docx]

**[Supplementary Information](https://static-content.springer.com/esm/art%3A10.1038%2Fs41467-020-17905-y/MediaObjects/41467_2020_17905_MOESM1_ESM.pdf)**

**Transplantation of hPSC-derived pericyte-like cells promotes functional recovery in ischemic stroke mice**

Sun et al.

**Supplementary Table 1. Primers used for qRT-PCR**

| **Gene** | **Direction** | **Sequences** |
| --- | --- | --- |
| *OCT4* | Forward | 5’ GAC AGG GGG AGG GGA GGA GCT AGG 3’ |
|  | Reverse | 5’ CTT CCC TCC AAC CAG TTG CCC CAA AC 3’ |
| *KLF4* | Forward | 5’ TGA TTG TAG TGC TTT CTG GCT GGG CTC C 3’ |
|  | Reverse | 5’ ACG ATC GTG GCC CCG GAA AAG GAC C 3’ |
| *p75* | Forward | 5’ CTG CCT GGA CAG CGT GAC GTT 3’ |
|  | Reverse | 5’ GCA GCG CCC AGT CGT CTC AT 3’ |
| *NANOG* | Forward | 5’ CAG CCC AGA TTC TTC CAC CAG TCC C 3’ |
|  | Reverse | 5’ CGG AAG CTT CCC AGT CGG GTT CAC C 3’ |
| *SOX10* | Forward | 5’ CTC AGC GGC TAC GAC TGG A 3’ |
|  | Reverse | 5’ GGC GCT TGT CAC TTT CGT TC 3’ |
| *AP2α* | Forward | 5’ AGG TCA ATC TCC CTA CAC GAG 3’ |
|  | Reverse | 5’ GGA GTA AGG ATC TTG CGA CTG G 3’ |
| *HOXA1* | Forward | 5’ TCC TGG AAT ACC CCA TAC TTA GC 3’ |
|  | Reverse | 5’ GCA CGA CTG GAA AGT TGT AAT CC 3’ |
| *HOXB1* | Forward | 5’ TGC CCT TCA GAA CCT AAC ACC C 3’ |
|  | Reverse | 5’ AGC TGC CTT GTG GTG AAG TTG G 3’ |
| *LHX5* | Forward | 5’ GCG CGT GGC ACA TCA AAT G 3’ |
|  | Reverse | 5’ GCC AAA GCG CCT GAA AAA GTC 3’ |
| *OTX2* | Forward | 5’ CAA AGT GAG ACC TGC CAA AAA GA 3’ |
|  | Reverse | 5’ TGG ACA AGG GAT CTG ACA GTG 3’ |
| *GAPDH* | Forward | 5’ CCC CTT CAT TGA CCT CAA CTA CA 3’ |
|  | Reverse | 5’ TTG CTG ATG ATC TTG AGG CTG T 3’ |
| *PDGFRβ* | Forward | 5’ TGC AGC ACC ACT CCG ACA AGC 3’ |
|  | Reverse | 5’ TCG CTC TCC CCG GTC AAG GAC 3’ |
| *NG2* | Forward | 5’ GTC TTT TGA GGC TGC CTG TC 3’ |
|  | Reverse | 5’ CTG TGT GAC CTG GAA GAG CA 3’ |
| *Caldesmon* | Forward | 5’ TCG ACC CAA CAA TAA CAG ATG C 3’ |
|  | Reverse | 5’ TCT CGT ATC TTT CTT GGC GAC T 3’ |
| *Vimentin* | Forward | 5’ TGC CGT TGA AGC TGC TAA CTA 3’ |
|  | Reverse | 5’ CCA GAG GGA GTG AAT CCA GAT TA 3’ |
| *Calponin* | Forward | 5’ TCA TCA AGG CCA TCA CCA AGT 3’ |
|  | Reverse | 5’ AGG GTG GAC TGC ACC TGT GTA 3’ |
| *ABCC9* | Forward | 5’ TCA ACC TGG TCC CTC ATG TCT 3’ |
|  | Reverse | 5’ CAG GAG AGC GAA TGT AAG AAT CC 3’ |
| *DLK1* | Forward | 5’ GCA CTG TGG GTA TCG TCT TCC 3’ |
|  | Reverse | 5’ CTC CCC GCT GTT GTA CTG AA 3’ |
| *KCNJ8* | Forward | 5’ CTC TTC GCT ATC ATG TGG TGG 3’ |
|  | Reverse | 5’ GAC CTG ACA TTA GTC ACA CAC AC 3’ |
| *ANPEP* | Forward | 5’ TTC AAC ATC ACG CTT ATC CAC C 3’ |
|  | Reverse | 5’ AGT CGA ACT CAC TGA CAA TGA AG 3’ |
| *COL1A1* | Forward | 5’ GTG CGA TGA CGT GAT CTG TGA 3’ |
|  | Reverse | 5’ CGG TGG TTT CTT GGT CGG T 3’ |
| *PDGFRA* | Forward | 5’ TTG AAG GCA GGC ACA TTT ACA 3’ |
|  | Reverse | 5’ GCG ACA AGG TAT AAT GGC AGA AT 3’ |
| *TBX18* | Forward | 5’ GAC GAT CTT TCT CCC ATC AAG C 3’ |
|  | Reverse | 5’ CTA TCT TCA GGC GAG TAA TCT GC 3’ |
| *LUM* | Forward | 5’ TAA CTG CCC TGA AAG CTA CCC 3’ |
|  | Reverse | 5’ GGA GGC ACC ATT GGT ACA CTT 3’ |
| *IFITM1* | Forward | 5’ GGG CCT TCT GGA TTC CGA G 3’ |
|  | Reverse | 5’ CGT GGG GTT GGT CAT CGT C 3’ |
| *COL1A2* | Forward | 5’ GAG CGG TAA CAA GGG TGA GC 3’ |
|  | Reverse | 5’ CTT CCC CAT TAG GGC CTC TC 3’ |
| *HOXC10* | Forward | 5’ ACA TGC CCT CGC AAT GTA ACT 3’ |
|  | Reverse | 5’ GAG AGG TAG GAC GGA TAG GTG 3’ |
| *HOXD10* | Forward | 5’ AGA CAG TTG GAC AGA TCC GAA 3’ |
|  | Reverse | 5’ CGA AAT GAG TTT GTT GCG CTT AT 3’ |
| *HOXA11* | Forward | 5’ TGC CAA GTT GTA CTT ACT ACG TC 3’ |
|  | Reverse | 5’ GTT GGA GGA GTA GGA GTA TGT CA 3’ |
| *HOXC11* | Forward | 5’ ATG TTT AAC TCG GTC AAC CTG G 3’ |
|  | Reverse | 5’ GCA TGT AGT AAG TGC AAC TGG G 3’ |
| *HOXC12* | Forward | 5’ ATG GGC GAG CAT AAT CTC CTG 3’ |
|  | Reverse | 5’ CGT GGG TAG GAC AGC GAA G 3’ |
| *MDK* | Forward | 5’ CGCGGTCGCCAAAAAGAAAG 3’ |
|  | Reverse | 5’ TACTTGCAGTCGGCTCCAAAC 3’ |
| *PTN* | Forward | 5’ GGA GCT GAG TGC AAG CAA AC 3’ |
|  | Reverse | 5’ CTC GCT TCA GAC TTC CAG TTC 3’ |

**Supplementary Table 2. List of antibodies used in FACS.**

| Antigen | Label | Dilution | Company | Cat. No. |
| --- | --- | --- | --- | --- |
| Anti-human CD13 | PE | 20 µl / test | BD Pharmingen | 555394 |
| Anti-human CD29 | PE | 20 µl / test | BD Pharmingen | 556049 |
| Anti-human CD44 | APC | 20 µl / test | BD Pharmingen | 559942 |
| Anti-human CD45 | PE-Cy^TM^7 | 5 µl / test | BD Pharmingen | 557748 |
| Anti-human CD57 | PE | 5 µl / test | BD Pharmingen | 560844 |
| Anti-human CD73 | FITC | 5 µl / test | BD Pharmingen | 561254 |
| Anti-human CD90 | APC | 5 µl / test | BD Pharmingen | 559869 |
| Anti-human CD105 | FITC | 5 µl / test | BD Pharmingen | 561443 |
| Anti-human CD140b | PE | 20 µl / test | BD Pharmingen | 558821 |
| Anti-human CD146 | BV421 | 5 µl / test | BD Horizon | 564325 |
| Anti-human CD271 | AF647 | 20 µl / test | BD Pharmingen | 560326 |
| Anti-Human CD140a | PE | 20 µl / test | BD Pharmingen | 556002 |
| Anti-Human CD248 | AF647 | 5 µl / test | BD Pharmingen | 564994 |
| Anti- Human NG2 | AF647 | 5 µl / test | BD Pharmingen | 562414 |
| Anti-Human Notch3 | PE | 5 µl / test | BD Pharmingen | 563593 |
| Anti-Human CD166 | PE | 20 µl / test | BD Pharmingen | 559263 |
| Anti-Human CD34 | PE-Cy^TM^7 | 5 µl / test | BD Pharmingen | 560710 |
| Anti-Human CD146 | PE | 20 µl / test | BD Pharmingen | 550315 |
| Anti-Human CD140a | APC | 5 µl / test | Biolegend | 323511 |

**Supplementary Table 3. Antibodies used in Immunofluorescence Staining**

| **Antigen** | **Host** | **Dilution** | **Company** | **Cat. No.** |
| --- | --- | --- | --- | --- |
| S100B | mouse | 1:500 | Novus Biologicals | NBP1-41373 |
| GFAP | rabbit | 1:1000 | Millipore | ab5804 |
| Tuj1 | mouse | 1:1000 | R&D System | MAB1195 |
| Peripherin | rabbit | 1:1000 | abcam | Ab123576 |
| p75 | rabbit | 1:500 | Promega | G3231 |
| p75 | mouse | 1:100 | Thermo Fisher Scientific | MA5-13314 |
| HNK1 | mouse | 1:300 | Sigma-Aldrich | c6680 |
| SOX9 | rabbit | 1:500 | EMD Millipore | AB5535 |
| SOX10 | rabbit | 1:500 | abcam | ab155279 |
| Ap2α | mouse | 1:200 | 3B5 | DSHB |
| HOXA1 | rabbit | 1:500 | abcam | ab208781 |
| Nestin | mouse | 1:200 | EMD Millipore | MAB5326 |
| Calponin | rabbit | 1:500 | abcam | ab46794 |
| NG2 | mouse | 1:200 | EMD Millipore | MAB5384 |
| NG2 | rabbit |  | EMD Millipore | MAB5320 |
| SOX10 | goat | 1:50 | Thermo Fisher Scientific | PA5-47001 |
| ZO-1 | rabbit | 1:100 | Thermo Fisher Scientific | 40-2200 |
| αSMA | mouse | 1:200 | abcam | ab5694 |
| PAX6 | rabbit | 1:350 | abcam | ab195045 |
| PDGFRβ | rabbit | 1:100 | abcam | ab32570 |
| Occludin | rabbit | 1:100 | Thermo Fisher Scientific | 71-1500 |
| CD31 | mouse | 1:50 | Abcam | ab64543 |
| CD31 | rat | 1:100 | BD bioscience | 550274 |
| GLUT1 | rabbit | 1:200 | Abcam | ab115730 |
| NFκB | mouse | 1:100 | Cell Signaling Technology | 6956 |
| Goat anti-mouse IgG Alexa 488 | goat | 1:500 | Invitrogen | A11001 |
| Goat anti-rabbit IgG Alexa 488 | goat | 1:500 | Invitrogen | A11008 |
| Goat anti-rabbit IgG Alexa 555 | goat | 1:500 | Invitrogen | A21428 |
| Goat anti-mouse IgG Alexa 555 | goat | 1:500 | Invitrogen | A21422 |
| Donkey anti-goat IgG Alexa 594 | donkey | 1:500 | Invitrogen | A11058 |

**Supplementary Table 4. Antibodies used in Western blotting**

| **Antigen** | **Host** | **Dilution** | **Company** | **Cat. No.** |
| --- | --- | --- | --- | --- |
| COL1A1 | rabbit | 1:1000 | Invitrogen | PA5-29569 |
| CD13/APN | rabbit | 1:1000 | Cell Signaling Technology | 32720 |
| IFITM1 | rabbit | 1:2000 | Abcam | ab233545 |
| PDGFRβ | rabbit | 1:1000 | Cell Signaling Technology | 3169 |
| Vimentin | rabbit | 1:1000 | Cell Signaling Technology | 5741S |
| β-actin | mouse | 1:1000 | Cell Signaling Technology | 3700 |
| MDK | rabbit | 1:1000 | Invitrogen | PA5-19640 |
| GAPDH | rabbit | 1:1000 | Cell Signaling Technology | 2118s |
| ZO-1 | rabbit | 1:500 | Thermo Fisher Scientific | 40-2200 |
| Occludin | rabbit | 1:500 | Thermo Fisher Scientific | 71-1500 |
| GLUT1 | rabbit | 1:1000 | Abcam | ab115730 |
| MMP9 | rabbit | 1:1000 | Abcam | ab38898 |
| Cyclophilin A | rabbit | 1:1000 | Abcam | ab42408 |
| anti-mouse IgG HRP-linked Ab | horse | 1:1000 | Cell Signaling Technology | 7076 |
| anti-rabbit IgG HRP-linked Ab | goat | 1:1000 | Cell Signaling Technology | 7074 |

**Supplemental Figure 1**


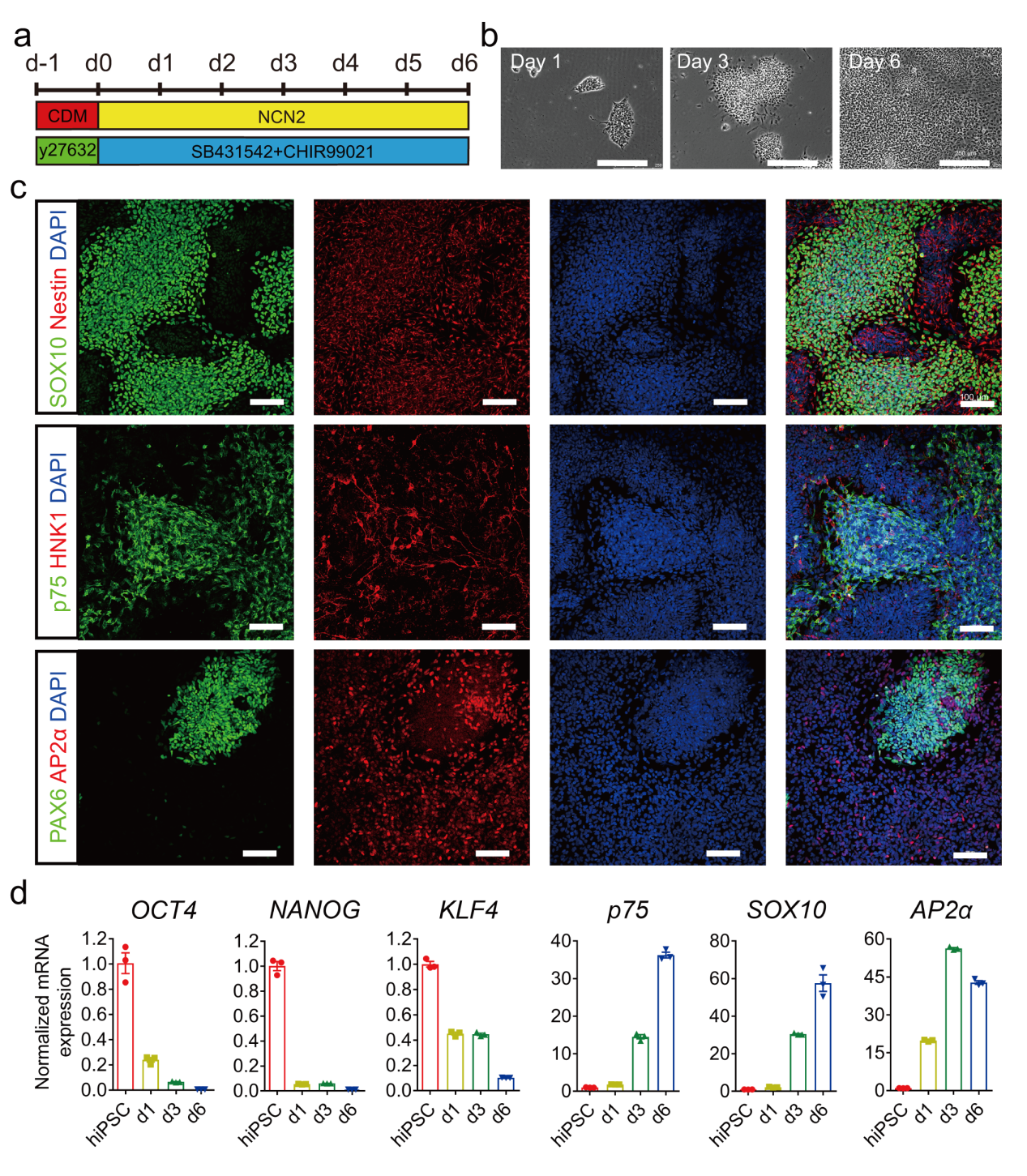


**Supplementary Figure 1 Generation of cranial** **neural crest cells (CNCs) from hiPSCs**

a. Schematic representation of the differentiation conditions used to generate cranial neural crest stem cells from hiPSCs;

b. Differentiated hiPSCs were observed under phase-contrast microscopy at day 1, day 3, and day 6. Scale bar: 250 μm.

c. Immunofluorescence staining was applied to detect the expression of neural crest markers (SOX10, Nestin, p75, HNK1, AP2α) and the neural epithelial cell marker PAX6 in differentiated cells. Scale bar: 100 μm.

d. qPCR was performed to analyze the expression of pluripotency genes (*OCT4*, *NANOG*, *KLF4*) and neural crest markers (*p75*, *SOX10*, *AP2*α) in undifferentiated and differentiated hiPSCs.

Graphs represent the individual data points, the mean ± SEM of three independent experiments. Confocal and bright field images are representative of n = 3 biological replicates. Source data are provided as a Source Data file.

**Supplemental Figure 2**


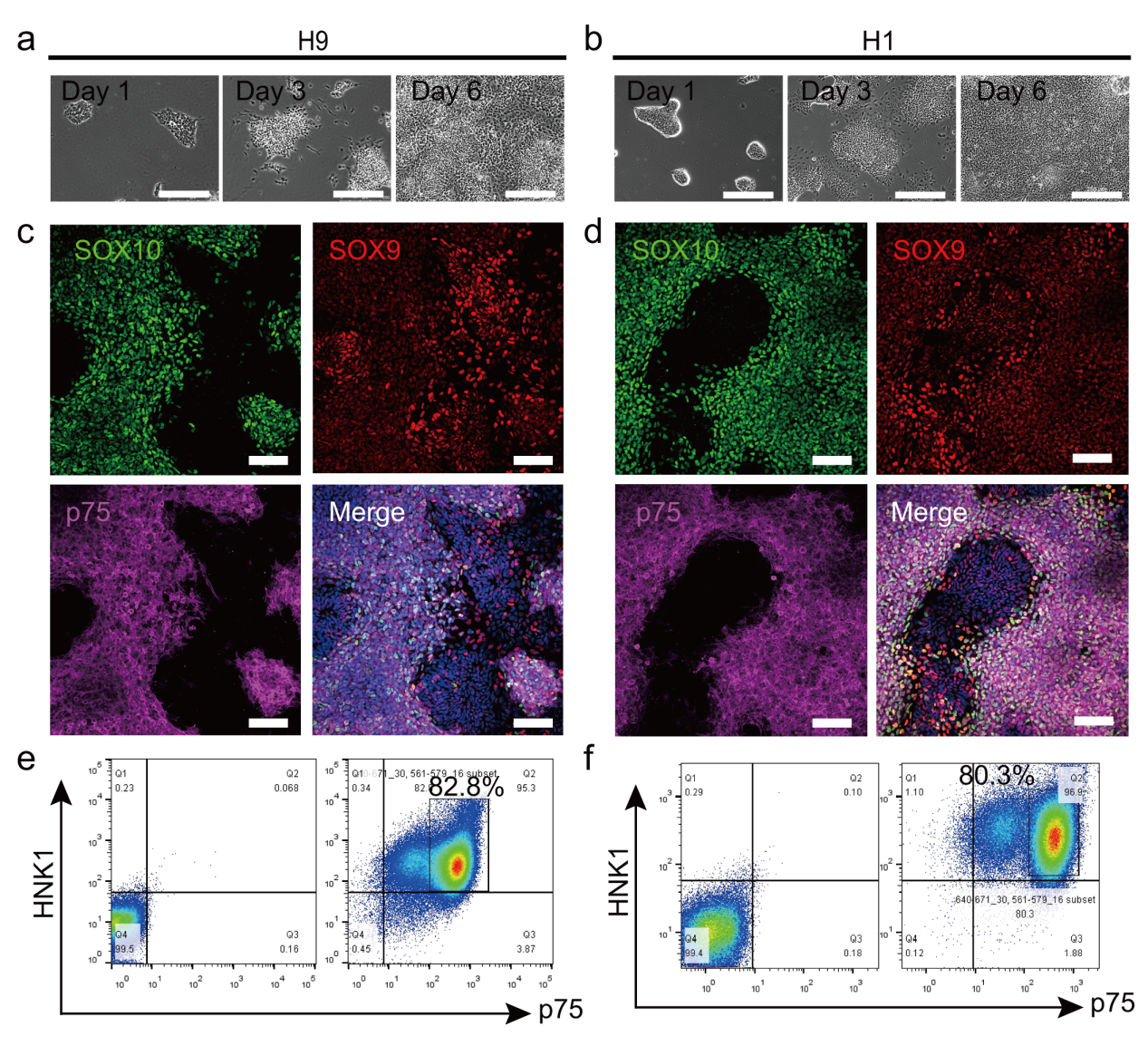


**Supplementary Figure 2 CNCs differentiated from hESCs**

a and b. The morphology of differentiated H9 cells (a) or H1 cells (b) was observed under phase-contrast microscopy at day 1, day 3, and day 6. Scale bar:250 μm.

c and d. Differentiated H9 cells (c) or H1 cells (d) at day 6 were analyzed for the expression of CNC markers by immunostaining. Scale bar: 100 μm.

e and f. p75^high^HNK1^+^ cranial CNCs from H9 (e) or H1 (f) cells were isolated by FACS.

FACS analysis, Confocal and bright field images are representative of n = 3 biological replicates.

**Supplemental Figure 3**


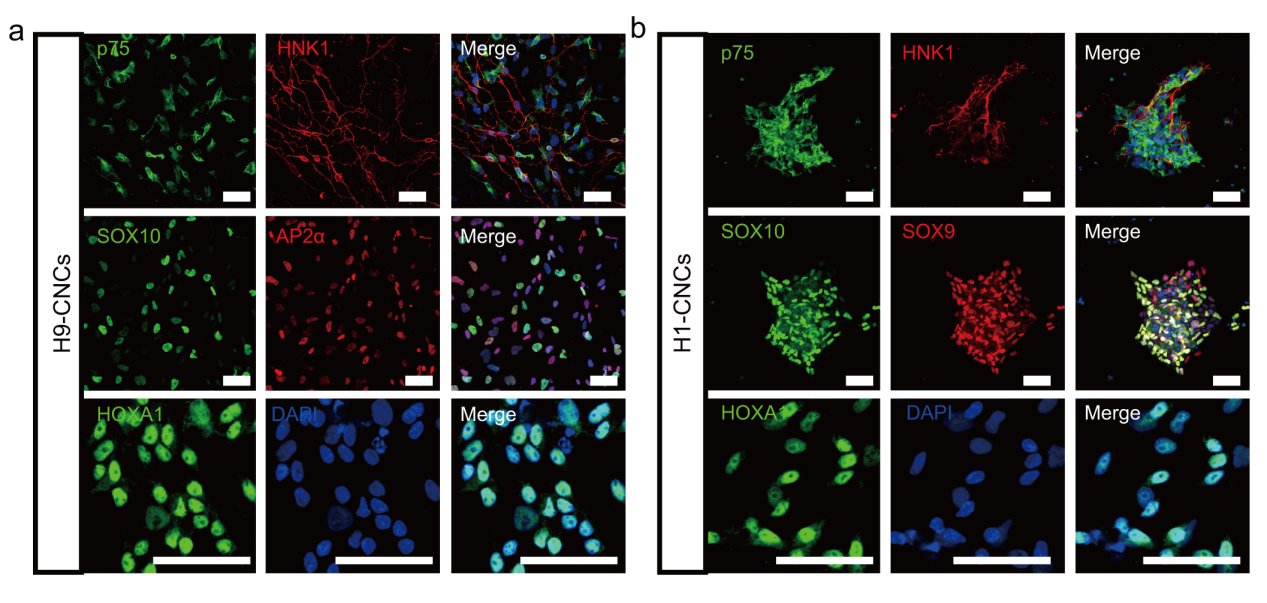


**Supplementary Figure 3 Characterization of CNCs derived from hESCs**

The expression of cranial CNC markers including p75, SOX10, SOX9, AP2α and HOXA1, in isolated H9-CNCs (a) or H1-CNCs (b) was assessed by immunostaining. Scale bar:50 μm.

Confocal images are representative of n = 3 biological replicates.

**Supplemental Figure 4**


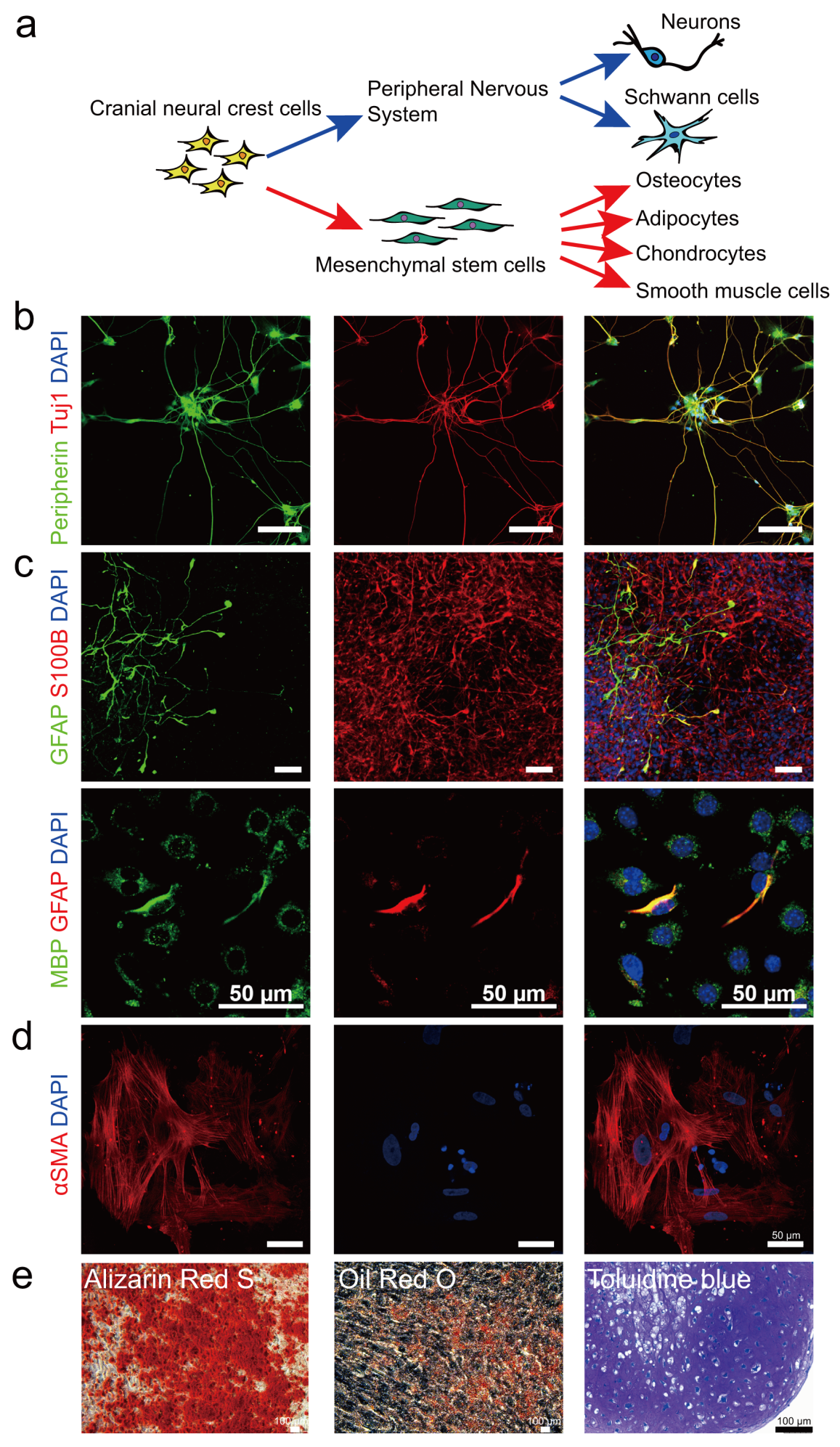


**Supplementary Figure 4 Multilineage differentiation of hiPSC-derived CNCs**

a. Schematic illustration of the experimental design.

b. Peripherin^+^/TUBB3^+^ peripheral neurons were detected in differentiated hiPSC-CNCs. Scale bar: 100 μm.

c. GFAP^+^, S100B^+^, or MBP^+^ Schwann cells were detected in differentiated hiPSC-CNCs. Scale bar: 100 μm (GFAP/S100B) and 50 μm (GFAP/MBP).

d. Anti-αSMA immunostaining was used to analyze the smooth muscle cell differentiation capacity of hiPSC-CNCs. Scale bar: 50 μm.

e. Alizarin Red S staining, Oil Red O staining, and toluidine blue staining were used to analyze the osteogenic, adipogenic, and chondrogenic differentiation capacity of hiPSC-CNCs, respectively. Scale bar: 100 μm.

Confocal images are representative of n = 3 biological replicates.

**Supplemental Figure 5**


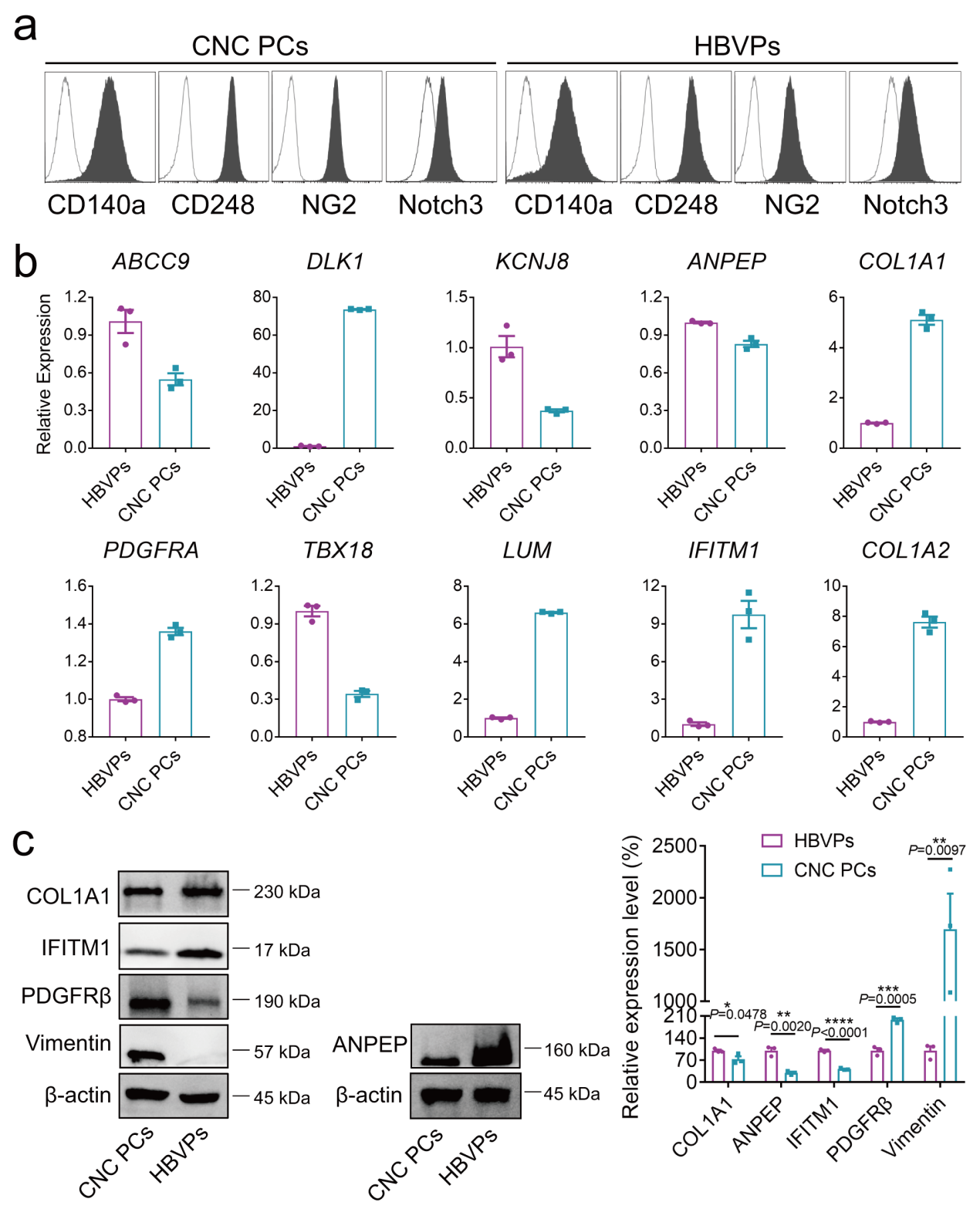


**Supplementary Figure 5 Comparison of phenotype between hPSC-CNC PCs and HBVPs**

a. FACS analysis for the surface marker expression of CNC PCs and HBVPs.

b. qPCR was used to analyze the expression of pericyte markers in CNC PCs and HBVPs.

c. Western blotting was performed to detect the expression of pericyte markers in CNC PCs and HBVPs. The relative levels of the proteins were determined and compared.

Graphs represent the individual data points, the mean ± SEM of three independent experiments. Blots are representative of n = 3 biological replicates. P value (*p < 0.05, **p < 0.01, ***p < 0.001, ****p < 0.0001) was calculated by two-tailed unpaired Student’s t-test. Source data are provided as a Source Data file.

**Supplemental Figure 6**


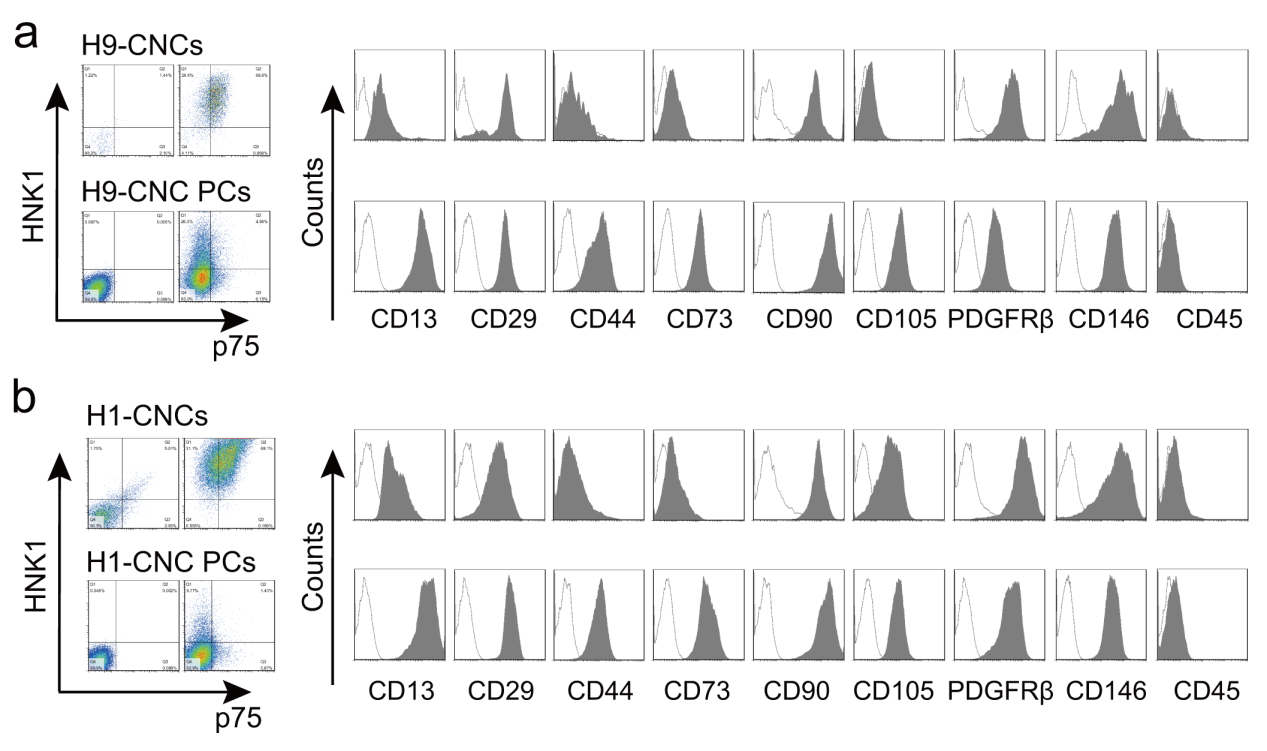


**Supplementary Figure 6 Flow cytometry analysis of hESC-CNC PCs**

The surface marker expression pattern of H9-CNC- (a) and H1-CNC-derived pericyte-like cells (b) was tested by FACS.

Images of FACS analysis are representative of n = 3 biological replicates.

**Supplemental Figure 7**


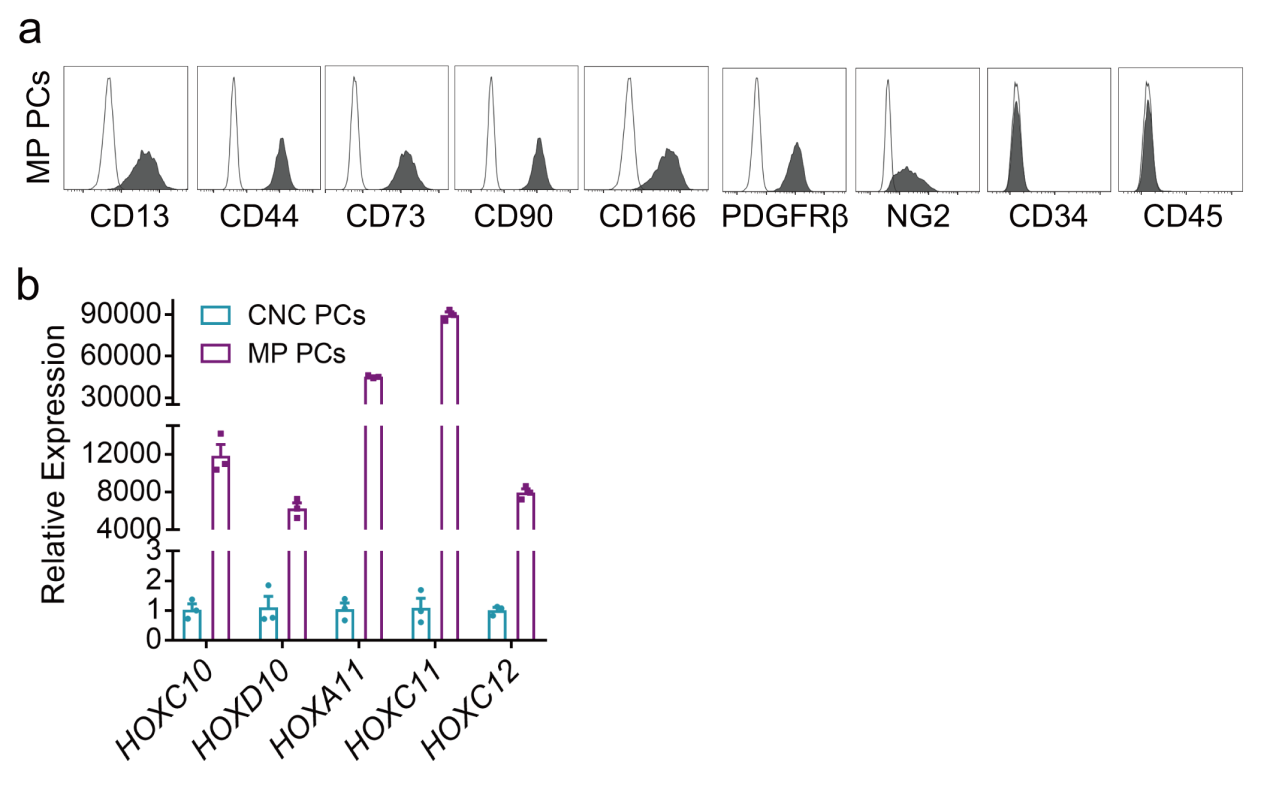


**Supplementary Figure 7 Characterization of mesoderm progenitor-derived pericyte-like cells (hPSC-MP PCs)**

a. FACS analysis for the surface marker expression of pericytes in hPSC-MP PCs.

b. qPCR was used to analyze the expression of posterior *HOX* genes in hPSC-CNC PCs and hPSC-MP PCs.

Graphs represent the individual data points, the mean ± SEM of three independent experiments. Images of FACS analysis are representative of n = 3 biological replicates. Source data are provided as a Source Data file.

**Supplemental Figure 8**


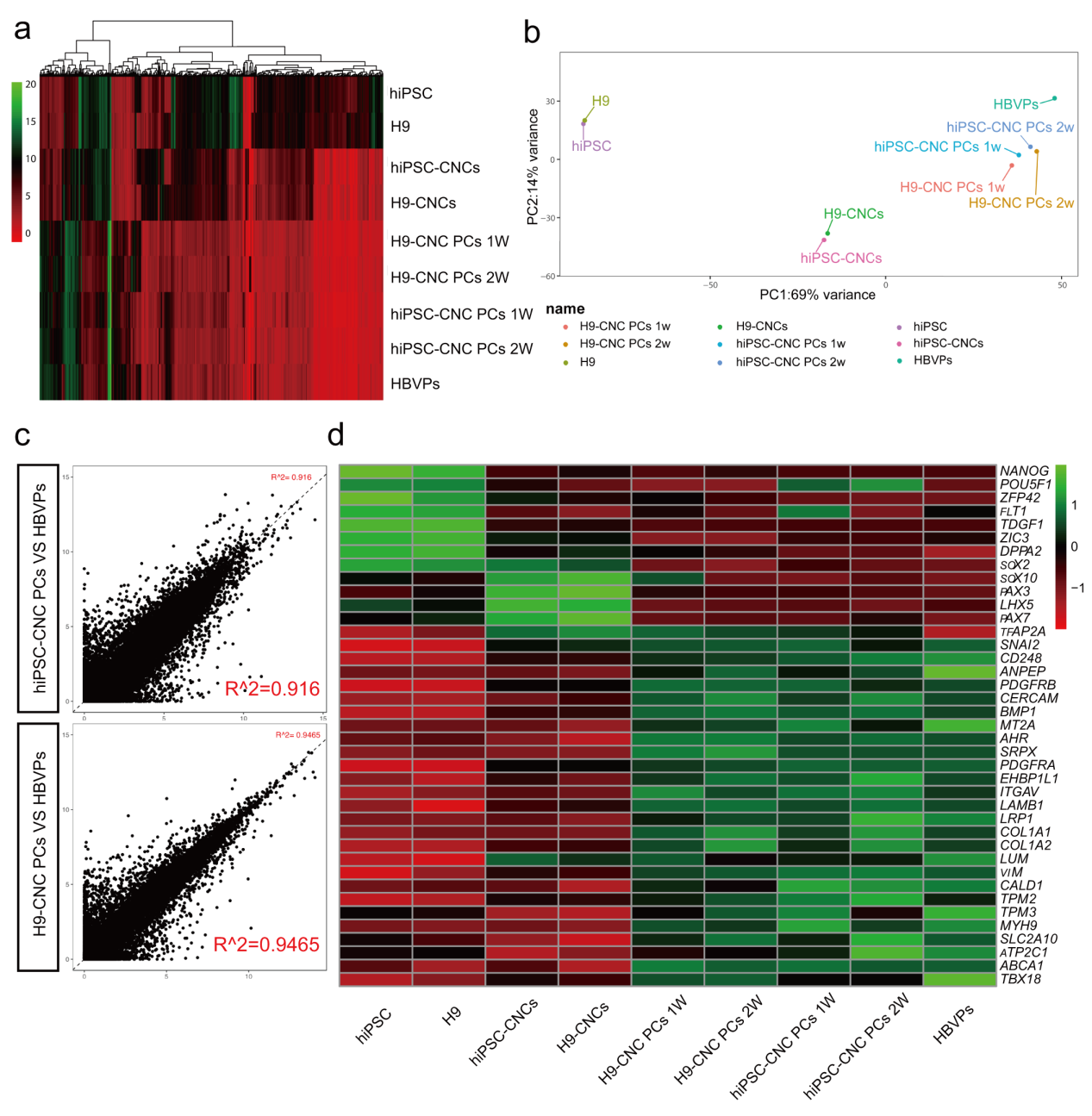


**Supplementary Figure 8 Global expression profiles of the cells during the pericyte differentiation of hPSCs**

a. Heat map of differential gene expression profiles of undifferentiated hPSCs, hPSC-CNCs, hPSC-CNC PCs (differentiation for 1 w or 2 w), and HBVPs.

b. Principal component analysis (PCA) was carried out to evaluate the similarities of the gene expression profiles between undifferentiated hPSCs, hPSC-CNCs and hPSC-CNC PCs.

c. Pearson’s correlation coefficients of pairwise comparisons were calculated for all expressed genes between hPSC-CNC PCs and HBVPs.

d. The enriched mRNA expression of hPSCs, hPSC-CNCs and hPSC-CNC PCs in RNA-Seq results was analyzed.

**Supplemental Figure 9**


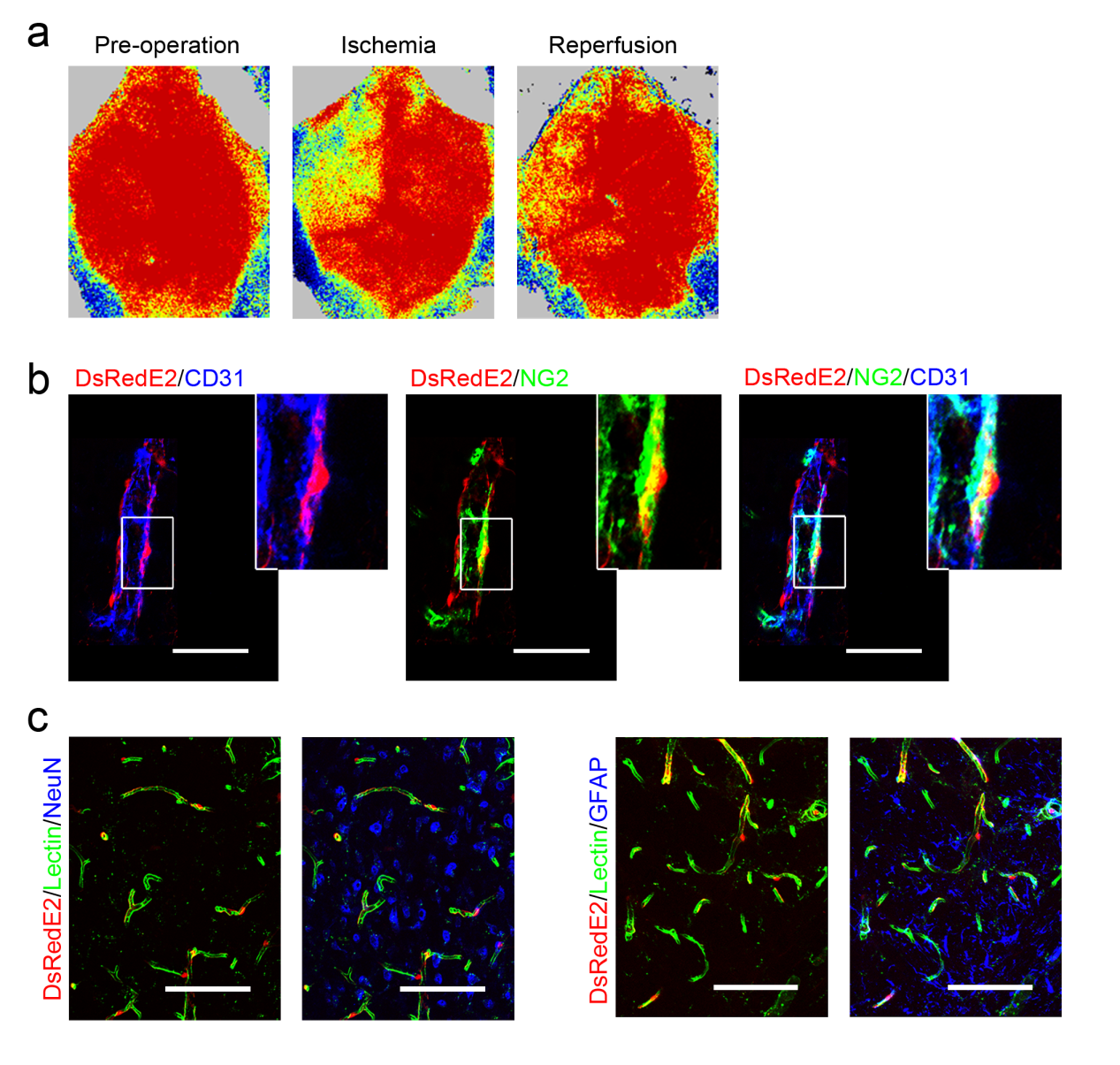


**Supplementary Figure 9 Laser Doppler flowmetry and in vivo characteristics of transplanted hPSC-CNC PCs**

a. Representative images of blood flow at 20min before surgery, the ischemic stage, and the reperfusion stage in tMCAO mice were evaluated by laser Doppler flowmetry.

b. Transplanted DsRedE2^+^ CNC PCs maintained the expression of pericyte-specific marker NG2 and were located near CD31^+^ endothelial cells. Scale bar: 20 μm.

c. Immunostaining showed that implanted DsRedE2^+^ CNC PCs did not express NeuN or GFAP. Scale bar: 100 μm.

Images are representative of n = 3 (a, b, c) biologically independent animals.

**Supplemental Figure 10**
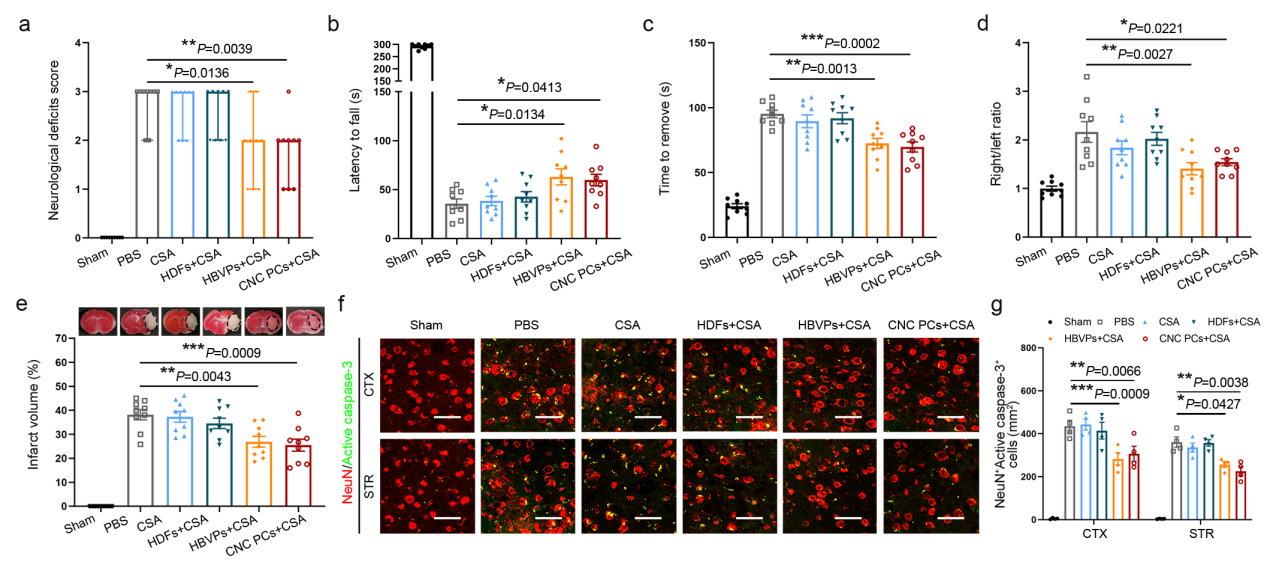


**Supplementary Figure 10 The assessment of neurological function and neuronal damage after treatment with PBS, CSA, HDFs+CSA, HBVPs+CSA and CNC PCs+CSA in stroke mice.**

a. Neurological deficit scores in different groups on day 3 were evaluated;

b. Motor coordination measurement from the rotarod test was rescued in tMCAO mice treated with HBVPs or CNC PCs;

c. The time to remove the sticker in the adhesive removal test was analyzed at 3 days after MCAO;

d. The right/left ratio in the corner test on day 3 was calculated;

e. Infarct volumes of different groups were determined at 7days poststroke and quantified on TTC (red)-stained coronal cerebral sections;

f. Fluorescent staining with NeuN (red) and active caspase-3 (green) showed neuronal apoptosis in the cortex (CTX) and striatum (STR) at day 3 after transplantation. Scale bar: 50 μm;

g. The bar graph shows the quantification of the percentage of active caspase-3^+^ neurons in different groups.

Graphs represent the individual data points, and data are presented as median ± 95% CI (a) or mean ± SEM (b, c, d, e, g). Behavior tests and infarct volume analysis are derived from n = 9 (a, b, c, d, e) biologically independent animals and confocal images are representative of n = 4 (f, g) independent animals. P value (*p < 0.05, **p < 0.01, ***p < 0.001, ****p < 0.0001) was calculated by one-way (a, b, c, d, e) or two-way ANOVA with Tukey post hoc test for multiple comparisons; (g). Source data are provided as a Source Data file.

**Supplemental Figure 11**
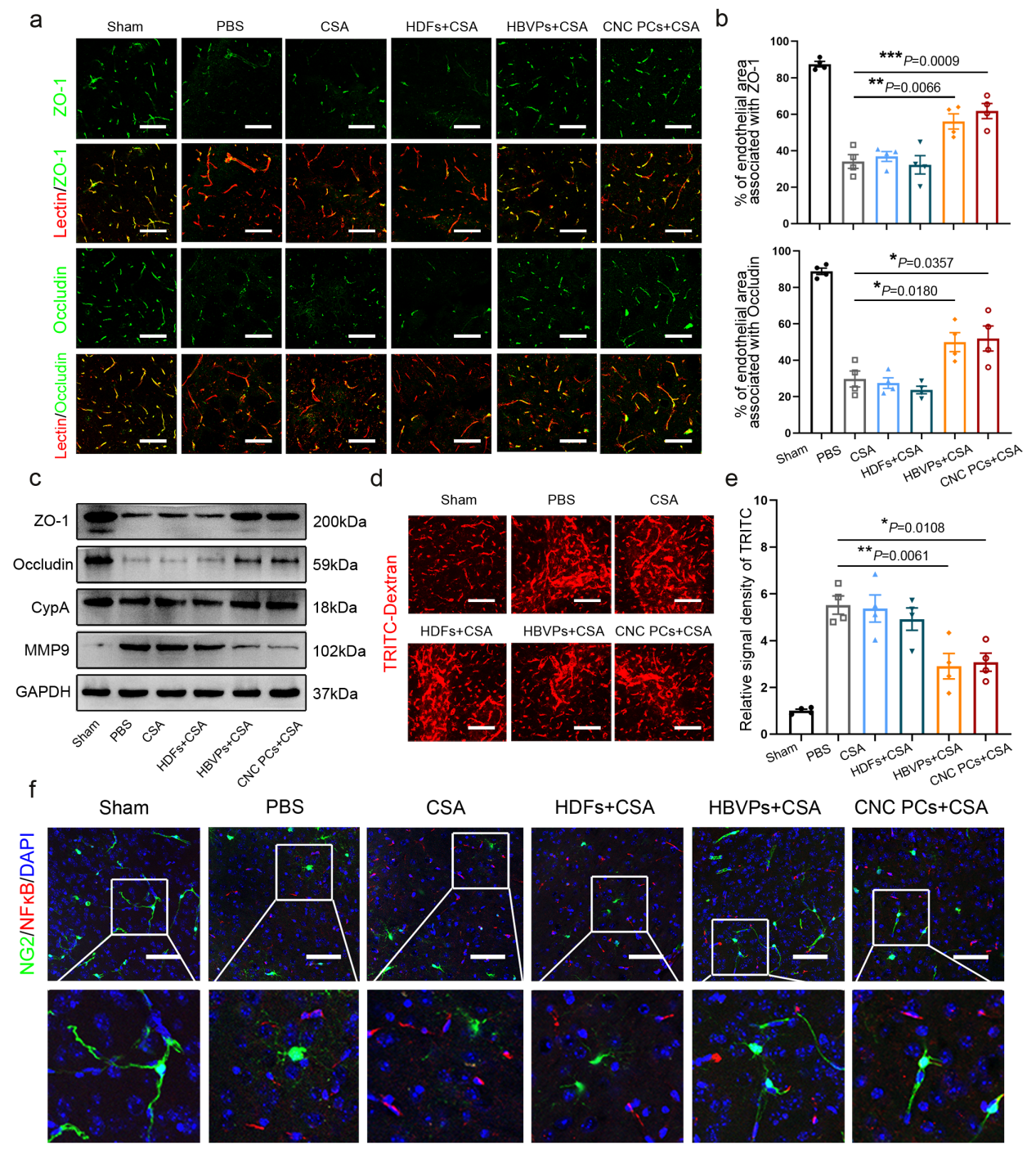


**Supplementary Figure 11 The recovery of the BBB function after treatment with PBS, CSA, HDFs+CSA, HBVPs+CSA and CNC PCs+CSA in stroke mice.**

a. Localization of ZO-1, Occludin, and lectin in the cerebral tissue of different groups was analyzed. Scale bar: 100 μm;

b. The quantification of cerebral tight junctions as the percentage of lectin-labeled endothelial area associated with ZO-1/Occludin;

c. Expression of ZO-1, Occludin, CypA, and MMP9 was evaluated in the ipsilateral hemisphere using microvessel western blotting 3 days after cell transplantation;

d. Representative images of TRITC-dextran tracer extravasation assay. Scale bar: 100 μm;

e. Quantification analysis of TRITC-dextran tracer extravasation assay;

f. Detection of NFκB nuclear translocation by immunofluorescence assay. Scale bar: 100 μm;

Graphs represent the individual data points, and data are presented as mean ± SEM. Confocal images and blots are representative of n = 4 (a, b, d, e, f) biologically independent animals. Immunoblotting images are representative of n = 3 (c) biological replicates. P value (*p < 0.05, **p < 0.01, ***p < 0.001, ****p < 0.0001) was calculated by one-way ANOVA with Tukey post hoc test for multiple comparisons; (b, e). Source data are provided as a Source Data file.

**Supplemental Figure 12**
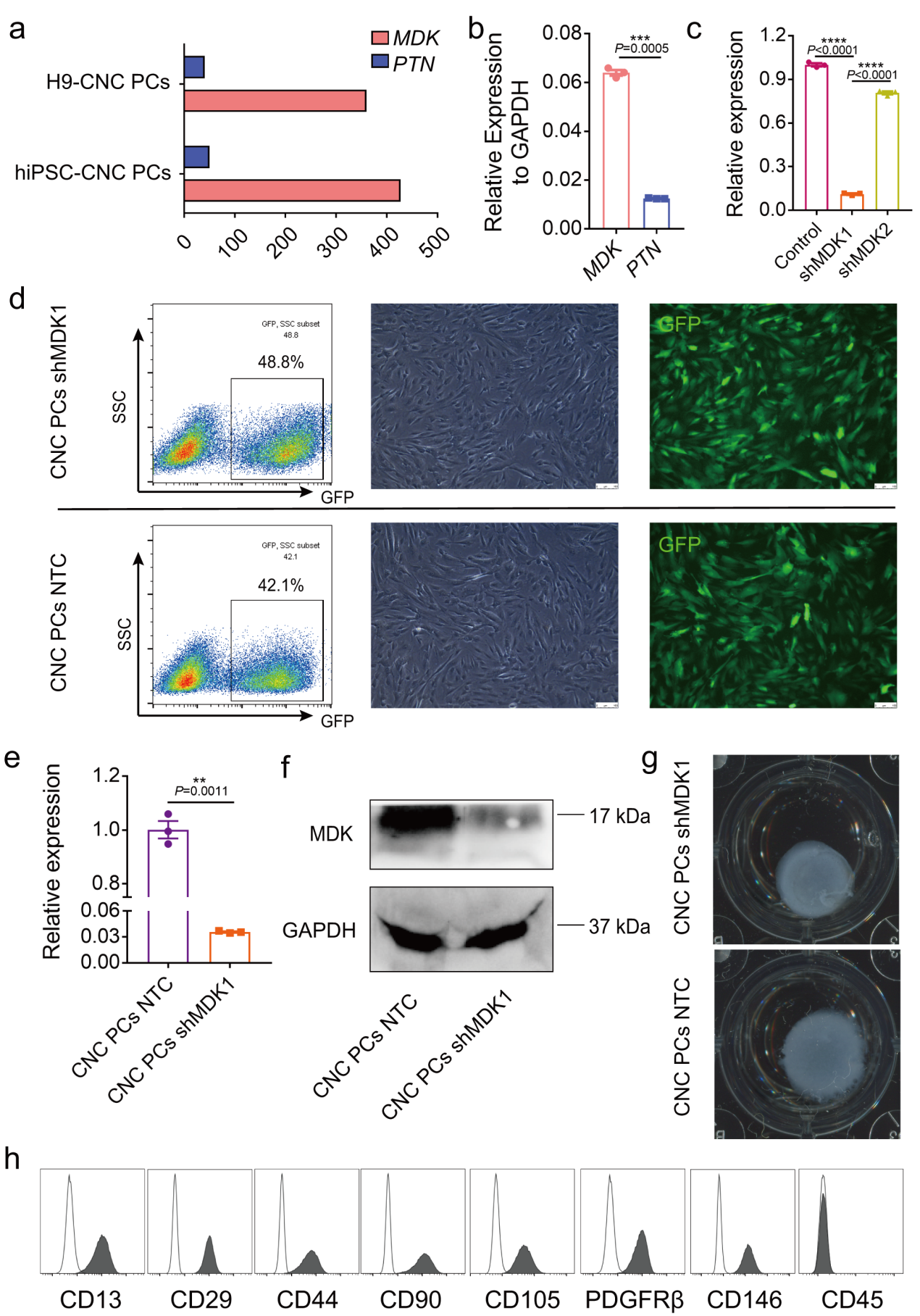


**Supplementary Figure 12 MDK knockdown in hPSC-CNC PCs**

a. The TPM values of *PTN* and *MDK* in hPSC-CNC PCs.

b. Detection of the mRNA levels of *PTN* and *MDK* in hPSC-CNC PCs by qPCR.

c. The knockdown efficiency of shMDK1 and shMDK2 was compared by qPCR.

d. Transduced GFP-expressing CNC PCs shMDK1 and CNC PCs NTC were enriched by FACS. Scale bar:100 μm.

e. qPCR was used to analyze the expression of *MDK* in CNC PCs shMDK1 and CNC PCs NTC.

f. Western blot was performed to detect MDK protein levels in CNC PCs shMDK1 and CNC PCs NTC.

g. A gel lattice contraction assay was applied to test the contractile properties of CNC PCs shMDK1 and CNC PCs NTC.

h. FACS analysis for the surface marker expression of pericytes in CNC PCs shMDK1.

Graphs represent the individual data points, the mean ± SEM of three independent experiments. Blots are representative of n = 3 biological replicates. P value (*p < 0.05, **p < 0.01, ***p < 0.001, ****p < 0.0001) was calculated by two-tailed unpaired Student’s t-test. Source data are provided as a Source Data file.

**Supplemental Figure 13**


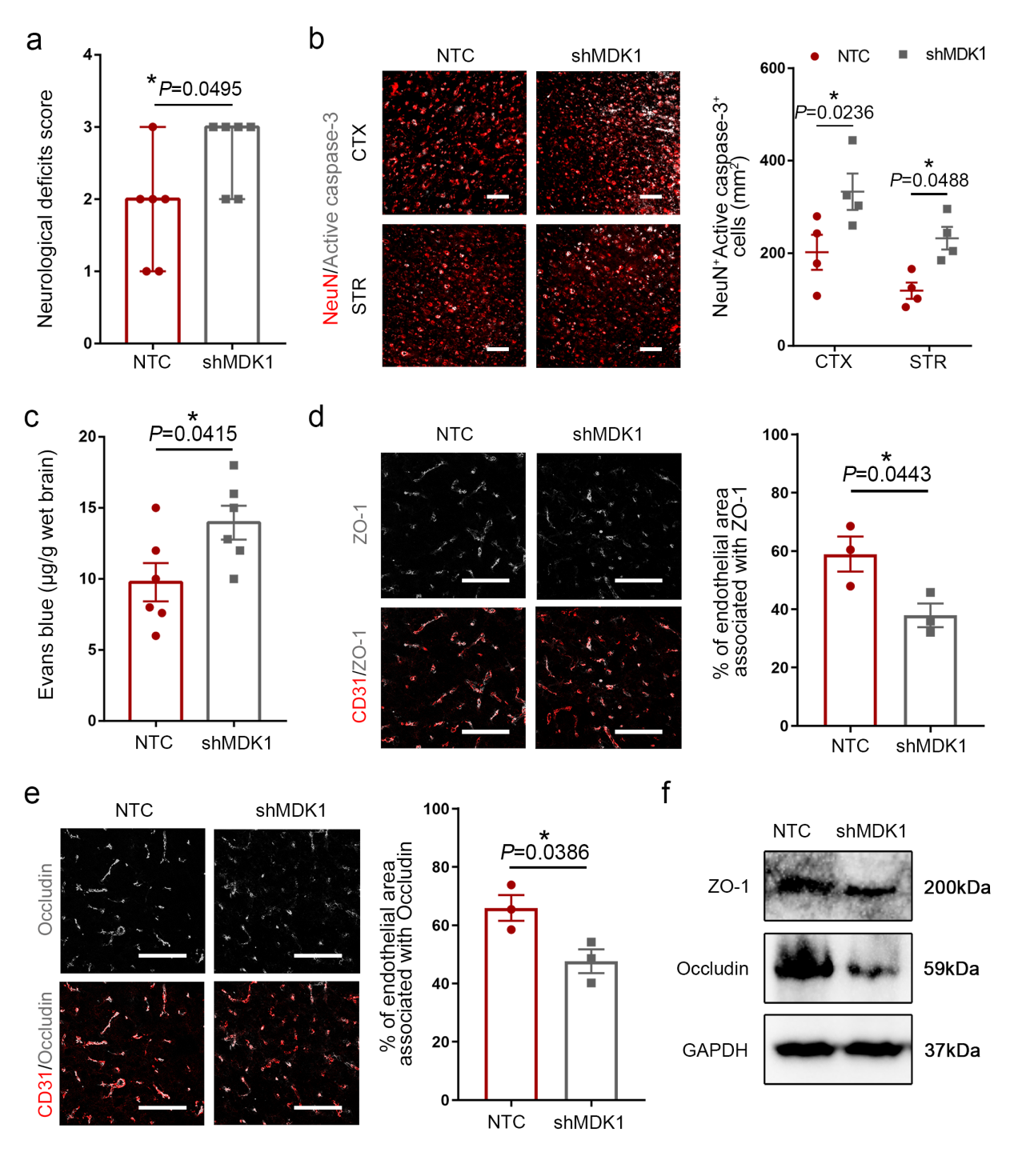


**Supplementary Figure 13 shMDK compromised the neuroprotective effects of hPSC-CNC PCs.**

a. MDK knockdown impaired the protective role of hPSC-CNC PCs in tMCAO-induced neurological deficits.

b. Confocal microscopy analysis and quantification of active caspase 3 immunodetection on NeuN^+^ (red) neurons. Scale bar: 50 μm.

c. Statistical analysis of Evans blue intensity by spectrofluorometry showed the increased BBB leakage in experimental mice of the shMDK group when compared to those in the NTC group.

d. The quantification of TJ coverage area was presented as the percentage of CD31-labeled endothelial area associated with ZO-1. Scale bar: 100 μm.

e. The quantification of TJ coverage area was presented as the percentage of CD31-labeled endothelial area associated with Occludin. Scale bar: 100 μm.

f. Representative western blotting images of TJ proteins ZO-1 and Occludin in the ipsilateral hemisphere.

Graphs represent the individual data points, and data are presented as median ± 95% CI (a) or mean ± SEM (b, c, d, e). Neurological scores and Evans blue extravasation assay are derived from n = 6 (a, c) biologically independent animals. FLICA staining images are representative of n = 4 (b) independent animals. Tight junction staining and immunoblotting images are representative of n = 3 (d, e, f) biological replicates. P value (*p < 0.05, **p < 0.01, ***p < 0.001, ****p < 0.0001) was calculated by two-tailed unpaired Student’s t-test (a, c, e) or two-way ANOVA with Tukey post hoc test for multiple comparisons; (b). Source data are provided as a Source Data file.

**Supplemental Figure 14**


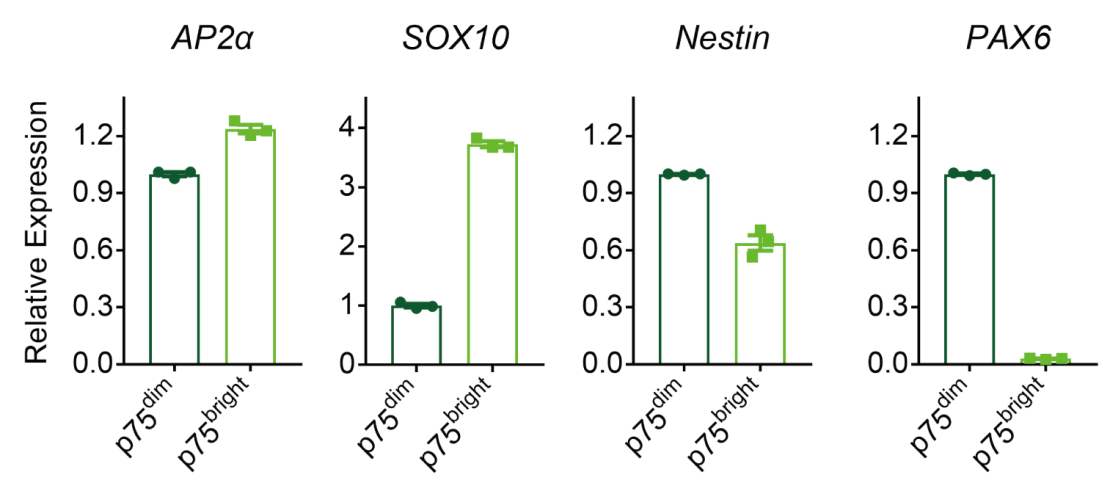


**Supplementary Figure 14 Detection of the mRNA levels of neural crest markers (*AP2α*, *SOX10*) and neural progenitor markers (*PAX6*, *Nestin*) in p75^bright^ and p75^dim^ CNCs by qPCR.**

Graphs represent the individual data points, the mean ± SEM of three independent experiments. Blots are representative of n = 3 biological replicates. Source data are provided as a Source Data file.

**Supplemental Figure 15**


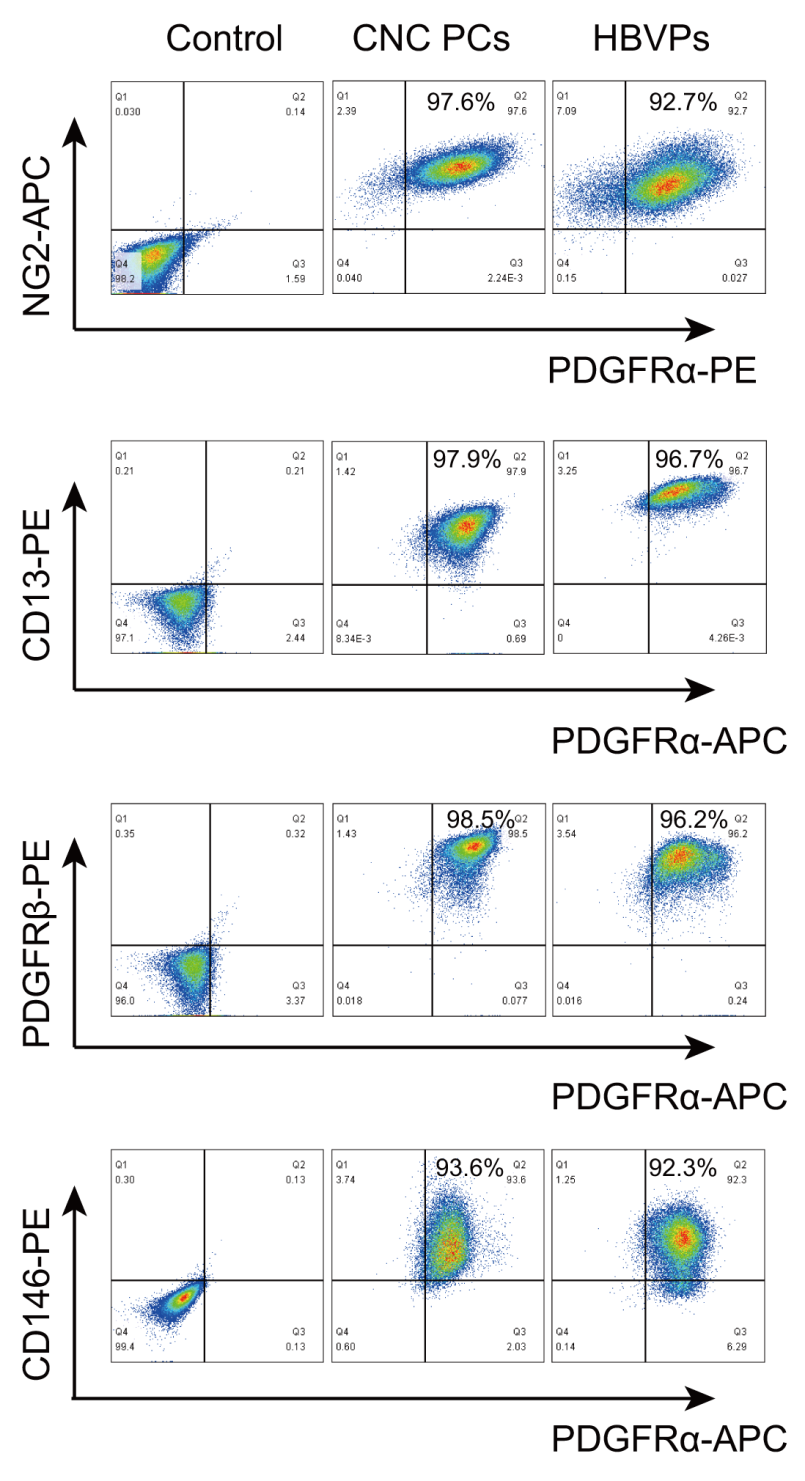


**Supplementary Figure 15 FACS analysis of surface marker expression in CNC PCs and HBVPs.**

Images of FACS analysis are representative of n = 3 biological replicates.

**Supplemental Figure 16**
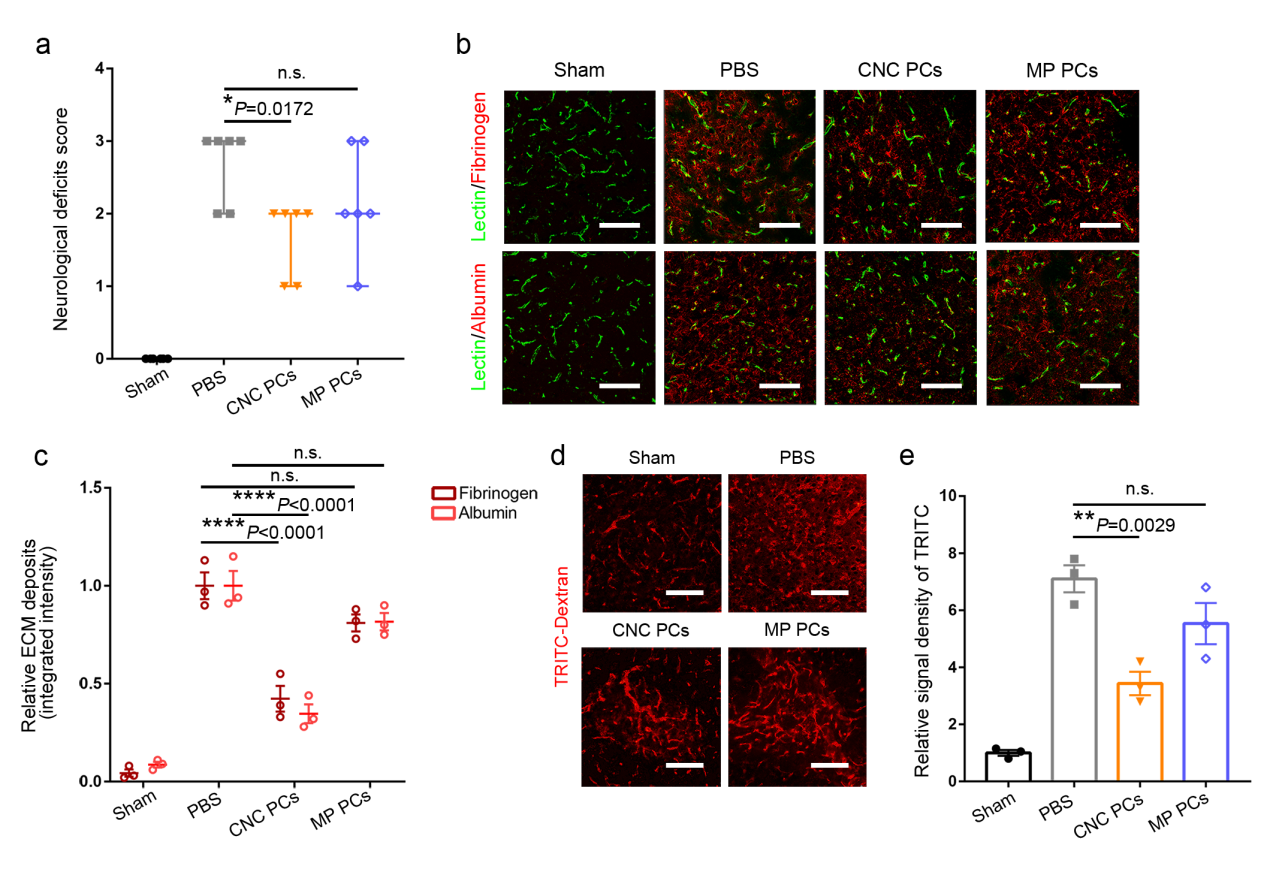


**Supplementary Figure 16 In vivo transplantation of hPSC-MP PCs in tMCAO mice.**

a. Neurological deficit scores were assessed at day 3 post-transplantation.

b. Representative confocal microscopy showing the extravascular fibrinogen (red) and albumin (red) leakage through lectin-labeled (green) capillaries. Scale bar: 100 μm.

c. Quantification of extravascular deposition of fibrinogen and albumin in the indicated experimental groups.

d. Representative immunostaining images of BBB integrity of the indicated mice as assessed by TRITC-dextran tracer extravasation assay. Scale bar: 100 μm.

e. Quantification of the extravasated exogenous tracer TRITC-dextran. The signal density of TRITC was measured by ImageJ and normalized to the sham group.

Graphs represent the individual data points, and data are presented as median ± 95% CI (a) mean ± SEM (c, e). Neurological scores evaluation are derived from n = 6 (a) biologically independent animals. Confocal images and blots are representative of n = 3 (b, c, d, e) independent animals. P value (*p < 0.05, **p < 0.01, ***p < 0.001, ****p < 0.0001) was calculated by one-way ANOVA (a, e) or two-way ANOVA with Tukey post hoc test for multiple comparisons; (c). Source data are provided as a Source Data file.

**Supplemental Figure 17**
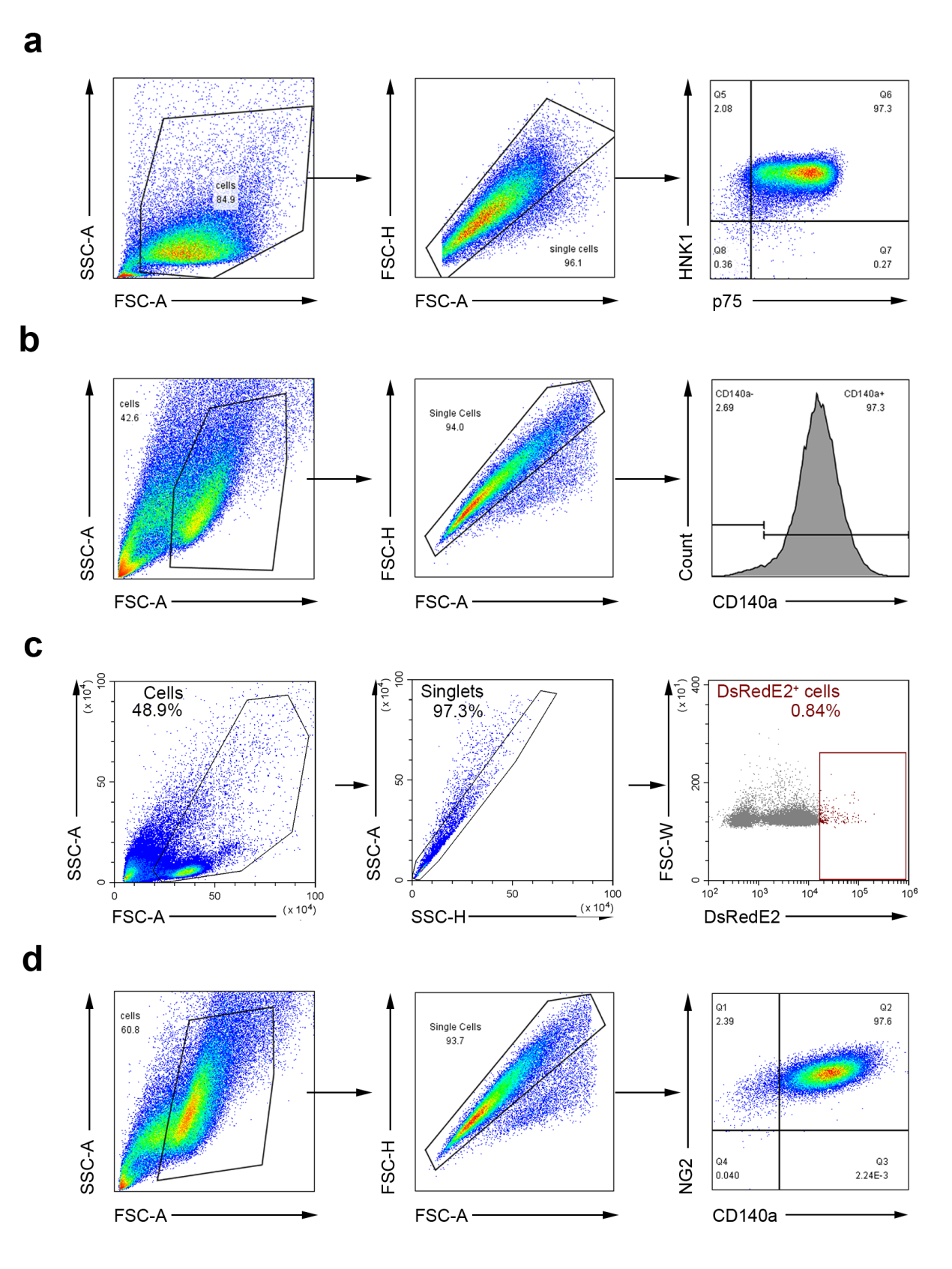


**Supplementary Figure 17 Gating strategies used for cell sorting.**

a. Gating strategies to sort p75^high^/HNK1^+^ CNCs.

b. Gating strategies to analyze the expression of pericyte-specific surface markers in CNC PCs and HBVPs.

c. Gating strategies to sort DsRedE2^+^ CNC PCs after transplantation.

d. Gating strategies to analyze the co-expression of pericyte-specific surface marker and fibroblast marker PDGFRα in CNC PCs and HBVPs.
