# Supplementary material for: Transplantation of hPSC-derived pericyte-like cells promotes functional recovery in ischemic stroke mice
Source: Nat Commun. 2020 Oct 15;11:5196. doi: 10.1038/s41467-020-19042-y (PMC7566513; doi:10.1038/s41467-020-19042-y)
Supplement: Supplementary file 3 — Reporting Summary [file 41467_2020_19042_MOESM3_ESM.pdf]

nature research

Corresponding author(s): Andy Peng Xiang, Weiqiang Li

Last updated by author(s): Aug 22, 2020

Reporting Summary

Nature Research wishes to improve the reproducibility of the work that we publish. This form provides structure for consistency and transparency in reporting. For further information on Nature Research policies, see Authors & Referees and the Editorial Policy Checklist .

Statistics

For all statistical analyses, confirm that the following items are present in the figure legend, table legend, main text, or Methods section.

n/a	Confirmed	
☐	☒	The exact sample size (n) for each experimental group/condition, given as a discrete number and unit of measurement
☐	☒	A statement on whether measurements were taken from distinct samples or whether the same sample was measured repeatedly
☐	☒	The statistical test(s) used AND whether they are one- or two-sided<br>*Only common tests should be described solely by name; describe more complex techniques in the Methods section.*
☒	☐	A description of all covariates tested
☒	☐	A description of any assumptions or corrections, such as tests of normality and adjustment for multiple comparisons
☐	☒	A full description of the statistical parameters including central tendency (e.g. means) or other basic estimates (e.g. regression coefficient) AND variation (e.g. standard deviation) or associated estimates of uncertainty (e.g. confidence intervals)
☐	☒	For null hypothesis testing, the test statistic (e.g. F, t, r) with confidence intervals, effect sizes, degrees of freedom and P value noted<br>*Give P values as exact values whenever suitable.*
☒	☐	For Bayesian analysis, information on the choice of priors and Markov chain Monte Carlo settings
☒	☐	For hierarchical and complex designs, identification of the appropriate level for tests and full reporting of outcomes
☐	☒	Estimates of effect sizes (e.g. Cohen's d, Pearson's r), indicating how they were calculated

Our web collection on statistics for biologists contains articles on many of the points above.

Software and code

Policy information about availability of computer code

Data collection	Q-PCR was performed using the LightCycler480 (Roche).<br>For flow cytometry, data were collected on CytoFLEX (Beckman Coulter) and Influx (BD Biosciences).<br>Immunofluorescence staining images were collected by LSM780 (Zeiss), LSM800 (Zeiss), Dragonfly 200 (Andor) and A1R N-SIM (Nikon).<br>The vascular network was photographed using BioTek Lionheart FX Automated Live Cell Imager (BioTek Instruments).<br>Luminescent signal was recorded using Infinite ® F200 pro microplate reader (Tecan).
Data analysis	GraphPad Prism 7 Software (GraphPad Software Inc) was used for statistical analysis.<br>Image J 1.52a Software (NIH) was used for analyzing the contractile experiment and immunostaining images.<br>CytExpert 2.0(Beckman Coulter) and FlowJo V 10.0 (Tree Star) were used to analyzed FACS data.<br>Ingenuity Pathways Analysis (IPA; Version: 52912811) software (Ingenuity Systems Inc.) was used for analyzing the RNA-Seq data.

For manuscripts utilizing custom algorithms or software that are central to the research but not yet described in published literature, software must be made available to editors/reviewers. We strongly encourage code deposition in a community repository (e.g. GitHub). See the Nature Research guidelines for submitting code & software for further information.

Data

Policy information about availability of data

All manuscripts must include a data availability statement. This statement should provide the following information, where applicable:

- Accession codes, unique identifiers, or web links for publicly available datasets
- A list of figures that have associated raw data
- A description of any restrictions on data availability

The authors declare that all data supporting the results in this study are available within the paper and its Supplementary Information. Raw data are available from the corresponding author upon reasonable request. The RNA-Seq data have been deposited in the GEO database, under accession number GSE132857 [https://

www.ncbi.nlm.nih.gov/geo/query/acc.cgi?acc=GSE132857]. Source data are provided with this paper.

Field-specific reporting

Please select the one below that is the best fit for your research. If you are not sure, read the appropriate sections before making your selection.

- [x] Life sciences
- [ ] Behavioural & social sciences
- [ ] Ecological, evolutionary & environmental sciences

For a reference copy of the document with all sections, see nature.com/documents/nr-reporting-summary-flat.pdf

Life sciences study design

All studies must disclose on these points even when the disclosure is negative.

Sample size

All sample size was indicated in figure legends. Sample size was chosen on our previous experience, experimental approach and standard practices in the field. No statistical methods were used to determine the sample size. The sample size is commonly acceptable and sufficient to determine significant differences in biological experiments (such as 8-9 independent animals for behavior tests and 3-4 biological replicates for biochemical assays including immunoblotting, immunofluorescence, FACS, etc.)

Data exclusions

No data were excluded from the analysis.

Replication

Experiments were performed successfully at least three times. All replication attempts were successfull.

Randomization

For experiment involving cellular and biological study three independent experiments have been performed, allocating randomly cells in experimental groups.
For animal study, animals were randomly put into cages and randomly assigned to experimental groups.

Blinding

All behavior tests were performed and analyzed by the researcher blinded to the group assignment. Quantification of immunofluorescent images were conducted by technicians blinded to the treatment assignment. For in vitro cell-based experiments, the investigators were not blinded to the treatment. As the group allocation and treatment were administered by the researcher collecting data, which make it difficult for blinding. For in vivo experiments other than behavior tests and immunostaining analysis, the researchers were not blinded to the group allocation. But we ensure that there was no human bias during data acquisition and analysis. Because most of the measurements were made using automated software, which is unsusceptible to operator bias.

Behavioural & social sciences study design

All studies must disclose on these points even when the disclosure is negative.

Study description

Briefly describe the study type including whether data are quantitative, qualitative, or mixed-methods (e.g. qualitative cross-sectional, quantitative experimental, mixed-methods case study).

Research sample

State the research sample (e.g. Harvard university undergraduates, villagers in rural India) and provide relevant demographic information (e.g. age, sex) and indicate whether the sample is representative. Provide a rationale for the study sample chosen. For studies involving existing datasets, please describe the dataset and source.

Sampling strategy

Describe the sampling procedure (e.g. random, snowball, stratified, convenience). Describe the statistical methods that were used to predetermine sample size OR if no sample-size calculation was performed, describe how sample sizes were chosen and provide a rationale for why these sample sizes are sufficient. For qualitative data, please indicate whether data saturation was considered, and what criteria were used to decide that no further sampling was needed.

Data collection

Provide details about the data collection procedure, including the instruments or devices used to record the data (e.g. pen and paper, computer, eye tracker, video or audio equipment) whether anyone was present besides the participant(s) and the researcher, and whether the researcher was blind to experimental condition and/or the study hypothesis during data collection.

Timing

Indicate the start and stop dates of data collection. If there is a gap between collection periods, state the dates for each sample cohort.

Data exclusions

If no data were excluded from the analyses, state so OR if data were excluded, provide the exact number of exclusions and the rationale behind them, indicating whether exclusion criteria were pre-established.

Non-participation

State how many participants dropped out/declined participation and the reason(s) given OR provide response rate OR state that no participants dropped out/declined participation.

Randomization

If participants were not allocated into experimental groups, state so OR describe how participants were allocated to groups, and if allocation was not random, describe how covariates were controlled.

Ecological, evolutionary & environmental sciences study design

All studies must disclose on these points even when the disclosure is negative.

Study description	*Briefly describe the study. For quantitative data include treatment factors and interactions, design structure (e.g. factorial, nested, hierarchical), nature and number of experimental units and replicates.*
Research sample	*Describe the research sample (e.g. a group of tagged Passer domesticus, all Stenocereus thurberi within Organ Pipe Cactus National Monument), and provide a rationale for the sample choice. When relevant, describe the organism taxa, source, sex, age range and any manipulations. State what population the sample is meant to represent when applicable. For studies involving existing datasets, describe the data and its source.*
Sampling strategy	*Note the sampling procedure. Describe the statistical methods that were used to predetermine sample size OR if no sample-size calculation was performed, describe how sample sizes were chosen and provide a rationale for why these sample sizes are sufficient.*
Data collection	*Describe the data collection procedure, including who recorded the data and how.*
Timing and spatial scale	*Indicate the start and stop dates of data collection, noting the frequency and periodicity of sampling and providing a rationale for these choices. If there is a gap between collection periods, state the dates for each sample cohort. Specify the spatial scale from which the data are taken*
Data exclusions	*If no data were excluded from the analyses, state so OR if data were excluded, describe the exclusions and the rationale behind them, indicating whether exclusion criteria were pre-established.*
Reproducibility	*Describe the measures taken to verify the reproducibility of experimental findings. For each experiment, note whether any attempts to repeat the experiment failed OR state that all attempts to repeat the experiment were successful.*
Randomization	*Describe how samples/organisms/participants were allocated into groups. If allocation was not random, describe how covariates were controlled. If this is not relevant to your study, explain why.*
Blinding	*Describe the extent of blinding used during data acquisition and analysis. If blinding was not possible, describe why OR explain why blinding was not relevant to your study.*

Did the study involve field work? ☐ Yes ☐ No

Field work, collection and transport

Field conditions	*Describe the study conditions for field work, providing relevant parameters (e.g. temperature, rainfall).*
Location	*State the location of the sampling or experiment, providing relevant parameters (e.g. latitude and longitude, elevation, water depth).*
Access and import/export	*Describe the efforts you have made to access habitats and to collect and import/export your samples in a responsible manner and in compliance with local, national and international laws, noting any permits that were obtained (give the name of the issuing authority, the date of issue, and any identifying information).*
Disturbance	*Describe any disturbance caused by the study and how it was minimized.*

Reporting for specific materials, systems and methods

We require information from authors about some types of materials, experimental systems and methods used in many studies. Here, indicate whether each material, system or method listed is relevant to your study. If you are not sure if a list item applies to your research, read the appropriate section before selecting a response.

Materials & experimental systems

n/a	Involved in the study	
☐	☒	Antibodies
☐	☒	Eukaryotic cell lines
☒	☐	Palaeontology
☐	☒	Animals and other organisms
☒	☐	Human research participants
☒	☐	Clinical data

Methods

n/a	Involved in the study	
☒	☐	ChIP-seq
☐	☒	Flow cytometry
☒	☐	MRI-based neuroimaging

Antibodies

Antibodies used

Detailed information about the antibodies used in this study are provided in Suppl. Tab. 2, Tab. 3 and Tab. 4.

Validation

All antibodies used in this study were commercially available and validation statement is on the manufacture's website.

Immunofluorescence

Anti-S100B Antibody (SA-12) (Novus Biologicals, Cat: NBP1-41373): Reaction with Human, Mouse, Rat, suitable for ICC/IF, IHC, this antibody was used in 3 citations.

Anti-Glial Fibrillary Acidic Protein (GFAP) Antibody (Sigma-Aldrich, Cat: AB5804): Reaction with Bovine, Canine, Human and Rat, suitable for ICC, IHC and IH(P), this antibody was used in 148 citations.

Anti-mouse Neuron-specific beta-III Tubulin Antibody (Clone TuJ-1, R&D System, Cat: MAB1195): Reaction with mouse, suitable for WB, IHC, IF-IC, this antibody was used in 148 citations.

Anti-Peripherin Antibody (Abcam, Cat: ab123576): Reaction with Mouse, Rat, Cat and Human, suitable for ICC/IF, this antibody was used in 0 citation.

Anti-human p75 pAb (Promega, Cat: G3231): Reaction with Human, suitable for IHC, IF and IP.

NGFR Monoclonal Antibody (Thermo Fisher Scientific, Cat: MA5-13314): Reaction with Cat, Ferret, Human, Non-human primary, Rabbit and Rat, suitable for Flow cytometry, ICC, IF, WB, IHC and IP, this antibody was used in 15 citations.

Anti-mouse HNK-1/N-CAM (CD57) Antibody (Sigma-Aldrich, Cat: c6680): Reaction with Rat, Human, Feline and Mouse, suitable for Flow cytometry and IHC, this antibody was used in 25 citations.

Anti-Sox9 Antibody (Sigma-Aldrich, Cat: AB5535): Reaction with chicken, rat, chicken, mouse, mouse, rat and human, suitable for ChIP, IHC, IF, IF-IC and WB, this antibody was used in 653 citations.

Recombinant Anti-Sox10 Antibody (Abcam, Cat: ab155279): Reaction with Mouse, Rat and Human, suitable for ICC/IF, IHC-FoFr, WB and Flow Cyt, this antibody was used in 33 citations.

Anti-human AP-2 alpha Antibody (DSHB, Cat: 3B5): Reaction with Chicken, Human, Mouse and Zebrafish, suitable for ChIP, IHC, IF, FFPE and WB, this antibody was used in 50 citations.

Anti-HOXA1 Antibody (Abcam, Cat: ab208781): Reaction with Mouse, Rat, Human and Zebrafish, suitable for ICC/IF, IP, IHC-P and WB, this antibody was used in 1 citation.

Anti-human Nestin Antibody (Clone 10C2, Sigma-Aldrich, Cat: MAB5326): Reaction with rat and mouse, suitable for ICC/IF, IHC, IHC-P and WB, this antibody was used in 386 citations.

Recombinant Anti-Calponin 1 Antibody (Abcam, Cat: ab46794): Reaction with Mouse, Rat, Human and Pig, suitable for WB, IHC-P and ICC/IF, this antibody was used in 161 citations.

Anti-NG2 Chondroitin Sulfate Proteoglycan Antibody (Clone 132.39, Sigma-Aldrich, Cat: MAB5384): Reaction with rat, human and mouse, suitable for WB, IHC, IF-IC and WB, this antibody was used in 107 citations.

SOX10 Polyclonal Antibody (Thermo Fisher Scientific, Cat: PA5-47001): Reaction with Human, suitable for ChIP, ICC, IF and WB, this antibody was used in 0 citation.

Recombinant ZO-1 Polyclonal Antibody (Thermo Fisher Scientific, Cat: 40-2200): Reaction with Dog, Human, Mouse and Rat, suitable for ICC, IF, IHC-F, WB, IHC, IHC-P and IP, this antibody was used in 210 citations.

Anti-alpha smooth muscle Actin Antibody (Abcam, Cat: ab5694): Reaction with Mouse, Chicken, Cow, Dog, Human and Pig, suitable for WB and IHC-P, this antibody was used in 1560 citations.

Recombinant Anti-PAX6 Antibody (Abcam, Cat: ab195045): Reaction with Mouse, Rat and Human, suitable for WB, IHC-P and ICC/IF, this antibody was used in 7 citations.

Recombinant Anti-PDGFR beta Antibody (Abcam, Cat: ab32570): Reaction with Mouse, Rat and Human, suitable for Flow Cyt, WB, IHC-P, ICC/IF and IP, this antibody was used in 167 citations.

Occludin Polyclonal Antibody (Thermo Fisher Scientific, Cat:71-1500): Reaction with Dog, Human and Rat, suitable for ELISA, ICC, IF, IHC, IP, WB, IHC-P and IHC-F, this antibody was used in 447 citations.

Anti-CD31 Antibody (Abcam, Cat: ab64543): Reaction with Rat, suitable for WB, IHC-Fr, Flow Cyt and ELISA, this antibody was used in 43 citations.

Anti-Mouse CD31 Antibody (Clone MEC 13.3, BD Pharmingen, Cat: 550274): Reaction with Mouse, suitable for IHC-P, IHC-F, Flow Cyt and IP, this antibody was used in 7 citations.

Recombinant Anti-Glucose Transporter GLUT1 Antibody (Abcam, Cat: ab115730): Reaction with Mouse, Rat and Human, suitable for ICC/IF, WB, IHC-P and Flow Cyt, this antibody was used in 75 citations.

NF-κB p65 (L8F6) Mouse mAb (Cell Signaling Technology, Cat: 6956): Reaction with Human, Mouse, Rat, Hamster, Monkey, Mink, Bovine, Dog and Pig, suitable for WB, IP, IHC, IF, Flow Cyt and ChIP, this antibody was used in 180 citations.

Alexa Fluor 488 Goat anti-Mouse IgG (H+L) Cross-Adsorbed Secondary Antibody (Invitrogen, Cat: A11001): Reaction with Mouse, suitable for ICC, WB, IP, IHC, IF, Flow Cyt, IHC-P and IHC-F, this antibody was used in 938 citations.

Alexa Fluor 488 Goat anti-Rabbit IgG (H+L) Cross-Adsorbed Secondary Antibody (Invitrogen, Cat: A11008): Reaction with Rabbit, suitable for ICC, WB, IP, IHC, IF, Flow Cyt, IHC-P and IHC-F, this antibody was used in 883 citations.

Alexa Fluor 555 Goat anti-Rabbit IgG (H+L) Cross-Adsorbed Secondary Antibody (Invitrogen, Cat: A21428): Reaction with Rabbit, suitable for ICC, WB, IP, IHC, IF, Flow Cyt, IHC-P and IHC-F, this antibody was used in 101 citations.

Alexa Fluor 594 Goat anti-Goat IgG (H+L) Cross-Adsorbed Secondary Antibody (Invitrogen, Cat: A11058): Reaction with Goat suitable for ICC, WB, IP, IHC, IF, Flow Cyt, IHC-P and IHC-F, this antibody was used in 80 citations.

Western blotting

COL1A1 Polyclonal Antibody (Invitrogen, Cat: PA5-29569): specific for mouse IgG, suitable for ICC, IF, IHC-P, IP and WB, Manufacturer website provides the datasheet "COL1A1 Polyclonal Antibody", The datasheet states this antibody was used in 3 citations.

CD13/APN (D6V1W) Rabbit mAb (Cell Signaling Technology, Cat: 32720):Reaction with human, mouse and rat, suitable for WB,

IP, IHC-P, IF-IC and F, Manufacturer website provides the datasheet “CD13/APN (D6V1W) Rabbit mAb”, The datasheet states this antibody was used in 1 citation.

Recombinant Anti-IFITM1 Antibody (Abcam, Cat: ab233545): specific for human IgG, suitable for WB, Flow Cyt, ICC/IF, IP and IHC-P, Manufacturer website provides the datasheet “Anti-IFITM1 antibody [EPR22620-12] ab233545”, The datasheet states this antibody was used in 0 citations.

PDGF Receptor β (28E1) Rabbit mAb (Cell Signaling Technology, Cat: 3169): Reaction with human, mouse and rat, suitable for WB, IP, IHC-P, IHC-F and IF-IC, Manufacturer website provides the datasheet “PDGF Receptor β (28E1) Rabbit mAb”, The datasheet states this antibody was used in 177 citations.

Vimentin (D21H3) XP® Rabbit mAb (Cell Signaling Technology, Cat: 5741S): Reaction with human, mouse, rat and monkey suitable for WB, IHC-P, IF-IC and F, Manufacturer website provides the datasheet “Vimentin (D21H3) XP® Rabbit mAb”, The datasheet states this antibody was used in 996 citations.

β-Actin (8H10D10) Mouse mAb (Cell Signaling Technology, Cat: 3700): Reaction with human, mouse, rat, hamster, monkey and dog, suitable for WB, IHC-P, IF-IC and F, Manufacturer website provides the datasheet “β-Actin (8H10D10) Mouse mAb”, The datasheet states this antibody was used in 1445 citations.

Midkine Polyclonal Antibody (Invitrogen, Cat: PA5-19640): specific for human IgG, suitable for WB, Manufacturer website provides the datasheet “Midkine Polyclonal Antibody”, The datasheet states this antibody was used in 1 citations.

GAPDH (14C10) Rabbit mAb (Cell Signaling Technology, Cat: 2118s): Reaction with human, mouse, rat, monkey, bovine and pig, suitable for WB, IHC-P, IF-IC and F, Manufacturer website provides the datasheet “GAPDH (14C10) Rabbit mAb”, The datasheet states this antibody was used in 3261 citations.

ZO-1 Polyclonal Antibody (Thermo Fisher Scientific, Cat: 40-2200): Reaction with human, mouse, rat and dog, suitable for WB, ICC, IF, IHC and IP, Manufacturer website provides the datasheet “ZO-1 Polyclonal Antibody”, The datasheet states this antibody was used in 210 citations.

Occludin Polyclonal Antibody (Thermo Fisher Scientific, Cat: 71-1500): Reaction with human, rat and dog, suitable for WB, ELISA, ICC, IF, IHC and IP, Manufacturer website provides the datasheet “Occludin Polyclonal Antibody”, The datasheet states this antibody was used in 18 citations.

Recombinant Anti-Glucose Transporter GLUT1 Antibody (Abcam, Cat: ab115730): Reaction with human, dog and mouse, suitable for IHC-Fr and WB, Manufacturer website provides the datasheet “Anti-Glucose Transporter GLUT1 antibody [EPR3915] ab115730”, The datasheet states this antibody was used in 75 citations.

Anti-MMP9 Antibody (Abcam, Cat: ab38898): Reaction with human, rat and mouse, suitable for ICC/IF, WB, IHC-P and Flow Cyt, Manufacturer website provides the datasheet “Anti-MMP9 antibody ab38898”, The datasheet states this antibody was used in 508 citations.

Anti-Cyclophilin A Antibody (Abcam, Cat: ab42408): Reaction with human, rat and mouse, suitable for ICC/IF, WB, Manufacturer website provides the datasheet “Anti-Cyclophilin A antibody ab42408”, The datasheet states this antibody was used in 5 citations.

Anti-mouse IgG, HRP-linked Antibody (Cell Signaling Technology, Cat: 7076): specific for mouse IgG, suitable for WB, Manufacturer website provides the datasheet “Anti-mouse IgG, HRP-linked Antibody”, The datasheet states this antibody was used in 3051 citations.

Anti-rabbit IgG, HRP-linked Antibody (Cell Signaling Technology, Cat: 7074): specific for rabbit IgG, suitable for WB, Manufacturer website provides the datasheet “Anti-mouse IgG, HRP-linked Antibody”, The datasheet states this antibody was used in 4146 citations.

Flow Cytometry

PE Mouse Anti-Human CD13 (Clone WM15, BD Pharmingen, Cat: 555394): Reactivity, Human; Application, Flow cytometry (Routinely Tested), this antibody was used in 5 citations.

PE Mouse Anti-Human CD29 (Clone HUTS-21, BD Pharmingen, Cat: 556049): Reactivity, Human; Application, Flow cytometry (Routinely Tested), this antibody was used in 5 citations.

APC Mouse Anti-Human CD44 (Clone G44-26, BD Pharmingen, Cat: 559942): Reactivity, Human; Application, Flow cytometry (Routinely Tested), this antibody was used in 8 citations.

PE-CyTM7 Mouse Anti-Human CD44 (Clone HI30, BD Pharmingen, Cat: 557748): Reactivity, Human; Application, Flow cytometry (Routinely Tested), this antibody was used in 9 citations.

PE Mouse Anti-Human CD57 (Clone HI30, BD Pharmingen, Cat: 560844): Reactivity, Human; Application, Flow cytometry (Routinely Tested), this antibody was used in 4 citations.

FITC Mouse Anti-Human CD73 (Clone AD2, BD Pharmingen, Cat: 561254): Reactivity, Human; Application, Flow cytometry (Routinely Tested), this antibody was used in 6 citations.

APC Mouse Anti-Human CD90 (Clone 5E10, BD Pharmingen, Cat: 559869): Reactivity, Human; Application, Flow cytometry (Routinely Tested), this antibody was used in 7 citations.

FITC Mouse Anti-Human CD105 (Clone 266, BD Pharmingen, Cat: 561443): Reactivity, Human; Application, Flow cytometry (Routinely Tested), this antibody was used in 5 citations.

PE Mouse Anti-Human CD140b (Clone 28D4, BD Pharmingen, Cat: 558821): Reactivity, Human; Application, Flow cytometry (Routinely Tested), this antibody was used in 5 citations.

BV421 Mouse Anti-Human CD146 (Clone P1H12, BD Horizon, Cat: 564325): Reactivity, Human; Application, Flow cytometry (Routinely Tested), this antibody was used in 7 citations.

AF647 Mouse Anti-Human CD271 (Clone C40-1457, BD Pharmingen, Cat: 560326): Reactivity, Human; Application, Flow cytometry (Routinely Tested), this antibody was used in 4 citations.

PE Mouse Anti-Human CD140a (Clone αR1, BD Pharmingen, Cat: 556002): Reactivity, Human; Application, Flow cytometry (Routinely Tested), this antibody was used in 5 citations.

AF647 Mouse Anti-Human CD248 (Clone B1/35, BD Pharmingen, Cat: 564994): Reactivity, Human; Application, Flow cytometry (Routinely Tested), this antibody was used in 4 citations.

AF647 Mouse Anti-Human Chondroitin Sulfate (Clone 9.2.27, BD Pharmingen, Cat: 562414): Reactivity, Human; Application, Flow

cytometry (Routinely Tested), this antibody was used in 6 citations.
PE Mouse Anti-Human Notch3 (Clone MHN3-21, BD Pharmingen, Cat: 563593): Reactivity, Human; Application, Flow cytometry (Routinely Tested), this antibody was used in 3 citations.
PE Mouse Anti-Human CD166 (Clone 3A6, BD Pharmingen, Cat: 559263): Reactivity, Human; Application, Flow cytometry (Routinely Tested), this antibody was used in 2 citations.
PE-CyTM7 Mouse Anti-Human CD34 (Clone 581, BD Pharmingen, Cat: 560710): Reactivity, Human; Application, Flow cytometry (Routinely Tested), this antibody was used in 6 citations.
PE Mouse Anti-Human CD146 (Clone P1H12, BD Pharmingen, Cat: 550315): Reactivity, Human; Application, Flow cytometry (Routinely Tested), this antibody was used in 4 citations.
APC anti-human CD140a (PDGFRα) Antibody (Clone 16A1, Biolegend, Cat: 323511): Reactivity, Human; Application, Flow cytometry (Routinely Tested), this antibody was used in 4 citations.

Eukaryotic cell lines

Policy information about cell lines

Cell line source(s)	HEK293FT cell line was from ATCC; HBVP, HBMEC and HASMC were from ScienCell Research Laboratories; H1 and H9 were from WiCell Research Institute.
Authentication	No cell lines were authenticated.
Mycoplasma contamination	All the cell lines have been tested negative for mycoplasma contamination by PCR methods.
Commonly misidentified lines (See ICLAC register)	No commonly misidentified cell lines were used.

Palaeontology

Specimen provenance	*Provide provenance information for specimens and describe permits that were obtained for the work (including the name of the issuing authority, the date of issue, and any identifying information).*
Specimen deposition	*Indicate where the specimens have been deposited to permit free access by other researchers.*
Dating methods	*If new dates are provided, describe how they were obtained (e.g. collection, storage, sample pretreatment and measurement), where they were obtained (i.e. lab name), the calibration program and the protocol for quality assurance OR state that no new dates are provided.*

☐ Tick this box to confirm that the raw and calibrated dates are available in the paper or in Supplementary Information.

Animals and other organisms

Policy information about studies involving animals; ARRIVE guidelines recommended for reporting animal research

Laboratory animals	8-10 weeks old male NOD-SCID mice and 8-12 weeks old male C57BL/6 mice were used. Mice were housed under standard specific-pathogen-free (SPF) conditions in a temperature-, humidity- and light cycle-controlled facility (20℃±2℃; 50%±10%; 12-h light/dark cycle) and free access to food and water.
Wild animals	No wild animals were used in this study.
Field-collected samples	No field-collected samples were used in this study.
Ethics oversight	All animal experiments were performed in accordance with protocols approved by the Ethical Committee of Sun Yat-sen University.

Note that full information on the approval of the study protocol must also be provided in the manuscript.

Human research participants

Policy information about studies involving human research participants

Population characteristics	*Describe the covariate-relevant population characteristics of the human research participants (e.g. age, gender, genotypic information, past and current diagnosis and treatment categories). If you filled out the behavioural & social sciences study design questions and have nothing to add here, write "See above."*
Recruitment	*Describe how participants were recruited. Outline any potential self-selection bias or other biases that may be present and how these are likely to impact results.*
Ethics oversight	*Identify the organization(s) that approved the study protocol.*

Note that full information on the approval of the study protocol must also be provided in the manuscript.

Clinical data

Policy information about clinical studies

All manuscripts should comply with the ICMJE guidelines for publication of clinical research and a completed CONSORT checklist must be included with all submissions.

Clinical trial registration	*Provide the trial registration number from ClinicalTrials.gov or an equivalent agency.*
Study protocol	*Note where the full trial protocol can be accessed OR if not available, explain why.*
Data collection	*Describe the settings and locales of data collection, noting the time periods of recruitment and data collection.*
Outcomes	*Describe how you pre-defined primary and secondary outcome measures and how you assessed these measures.*

ChIP-seq

Data deposition

- [ ] Confirm that both raw and final processed data have been deposited in a public database such as GEO.
- [ ] Confirm that you have deposited or provided access to graph files (e.g. BED files) for the called peaks.

Data access links<br>*May remain private before publication.*	*For "Initial submission" or "Revised version" documents, provide reviewer access links. For your "Final submission" document, provide a link to the deposited data.*
Files in database submission	*Provide a list of all files available in the database submission.*
Genome browser session<br>(e.g. UCSC)	*Provide a link to an anonymized genome browser session for "Initial submission" and "Revised version" documents only, to enable peer review. Write "no longer applicable" for "Final submission" documents.*

Methodology

Replicates	*Describe the experimental replicates, specifying number, type and replicate agreement.*
Sequencing depth	*Describe the sequencing depth for each experiment, providing the total number of reads, uniquely mapped reads, length of reads and whether they were paired- or single-end.*
Antibodies	*Describe the antibodies used for the ChIP-seq experiments; as applicable, provide supplier name, catalog number, clone name, and lot number.*
Peak calling parameters	*Specify the command line program and parameters used for read mapping and peak calling, including the ChIP, control and index files used.*
Data quality	*Describe the methods used to ensure data quality in full detail, including how many peaks are at FDR 5% and above 5-fold enrichment.*
Software	*Describe the software used to collect and analyze the ChIP-seq data. For custom code that has been deposited into a community repository, provide accession details.*

Flow Cytometry

Plots

Confirm that:

- [x] The axis labels state the marker and fluorochrome used (e.g. CD4-FITC).
- [x] The axis scales are clearly visible. Include numbers along axes only for bottom left plot of group (a 'group' is an analysis of identical markers).
- [x] All plots are contour plots with outliers or pseudocolor plots.
- [x] A numerical value for number of cells or percentage (with statistics) is provided.

Methodology

Sample preparation	Cells were dissociated with Accutase and single-cell suspensions were prepared in FACS buffer. Cell-surface staining was completed using the antibodies outlined in Supplementary Table 2.
Instrument	Samples were run on a CytoFLEX (Beckman Coulter) and a Influx (BD Biosciences).
Software	The data were analyzed using the CytExpert and Flow Jo software.

Cell population abundance

At least 20,000 cells were collected for each sample.

Gating strategy

A gate is drawn around the cells. Single cells are determined with the area and the height of the forward scatter (FSC). The analysis was carried out in this gate.

☒ Tick this box to confirm that a figure exemplifying the gating strategy is provided in the Supplementary Information.

Magnetic resonance imaging

Experimental design

Design type

Indicate task or resting state; event-related or block design.

Design specifications

Specify the number of blocks, trials or experimental units per session and/or subject, and specify the length of each trial or block (if trials are blocked) and interval between trials.

Behavioral performance measures

State number and/or type of variables recorded (e.g. correct button press, response time) and what statistics were used to establish that the subjects were performing the task as expected (e.g. mean, range, and/or standard deviation across subjects).

Acquisition

Imaging type(s)

Specify: functional, structural, diffusion, perfusion.

Field strength

Specify in Tesla

Sequence & imaging parameters

Specify the pulse sequence type (gradient echo, spin echo, etc.), imaging type (EPI, spiral, etc.), field of view, matrix size, slice thickness, orientation and TE/TR/flip angle.

Area of acquisition

State whether a whole brain scan was used OR define the area of acquisition, describing how the region was determined.

Diffusion MRI ☐ Used ☐ Not used

Preprocessing

Preprocessing software

Provide detail on software version and revision number and on specific parameters (model/functions, brain extraction, segmentation, smoothing kernel size, etc.).

Normalization

If data were normalized/standardized, describe the approach(es): specify linear or non-linear and define image types used for transformation OR indicate that data were not normalized and explain rationale for lack of normalization.

Normalization template

Describe the template used for normalization/transformation, specifying subject space or group standardized space (e.g. original Talairach, MNI305, ICBM152) OR indicate that the data were not normalized.

Noise and artifact removal

Describe your procedure(s) for artifact and structured noise removal, specifying motion parameters, tissue signals and physiological signals (heart rate, respiration).

Volume censoring

Define your software and/or method and criteria for volume censoring, and state the extent of such censoring.

Statistical modeling & inference

Model type and settings

Specify type (mass univariate, multivariate, RSA, predictive, etc.) and describe essential details of the model at the first and second levels (e.g. fixed, random or mixed effects; drift or auto-correlation).

Effect(s) tested

Define precise effect in terms of the task or stimulus conditions instead of psychological concepts and indicate whether ANOVA or factorial designs were used.

Specify type of analysis: ☐ Whole brain ☐ ROI-based ☐ Both

Statistic type for inference
(See Eklund et al. 2016)

Specify voxel-wise or cluster-wise and report all relevant parameters for cluster-wise methods.

Correction

Describe the type of correction and how it is obtained for multiple comparisons (e.g. FWE, FDR, permutation or Monte Carlo).

Models & analysis

n/a	Involved in the study
☐	☐ Functional and/or effective connectivity
☐	☐ Graph analysis
☐	☐ Multivariate modeling or predictive analysis

Functional and/or effective connectivity	*Report the measures of dependence used and the model details (e.g. Pearson correlation, partial correlation, mutual information).*
Graph analysis	*Report the dependent variable and connectivity measure, specifying weighted graph or binarized graph, subject- or group-level, and the global and/or node summaries used (e.g. clustering coefficient, efficiency, etc.).*
Multivariate modeling and predictive analysis	*Specify independent variables, features extraction and dimension reduction, model, training and evaluation metrics.*
