# Supplementary material for: Transplantation of hPSC-derived pericyte-like cells promotes functional recovery in ischemic stroke mice
Source: Nat Commun. 2020 Oct 15;11:5196. doi: 10.1038/s41467-020-19042-y (PMC7566513; doi:10.1038/s41467-020-19042-y)
Supplement: Supplementary file 4 — Description of Additional Supplementary Files [file 41467_2020_19042_MOESM4_ESM.pdf]

## Description of Additional Supplementary Files

Supplementary Movie 1 Cord formation of HBVPs

Supplementary Movie 2 Cord formation of CNC PCs

Supplementary Movie 3 Location of transplanted CNC PCs
